# Effectiveness and safety of vedolizumab in real-world studies in Asian patients with inflammatory bowel disease: a systematic review and meta-analysis

**DOI:** 10.1186/s12876-026-04975-3

**Published:** 2026-06-12

**Authors:** Yubei Gu, Xiaoyan Zhang, Lifei Gu, Duowu Zou

**Affiliations:** 1 Department of Gastroenterology, Ruijin Hospital Affiliated to Shanghai Jiao Tong University School of Medicine, Shanghai, China; 2 Medical Affairs, Takeda (China) Holdings Co., Ltd, Shanghai, China

**Keywords:** Vedolizumab, Inflammatory Bowel Disease, Crohn's Disease, Ulcerative Colitis, Asia

## Abstract

**Background:**

To evaluate the real-world effectiveness and safety of vedolizumab (VDZ) in Asian patients with IBD, including Crohn’s disease (CD) and Ulcerative colitis (UC).

**Methods:**

We systematically searched PubMed, EMBASE, the Cochrane Library, Web of Science, CBM, CNKI, Wanfang, and VIP for real-world studies assessing intravenous VDZ in Asian IBD patients. Primary outcomes were clinical remission, response and endoscopic remission. Secondary outcomes encompassed corticosteroid-free clinical remission, endoscopic response, complete endoscopic remission, C-reactive protein remission, faecal calprotectin normalization, treatment persistence rate and safety variables. Meta-analysis was performed for CD and UC across induction (6–14 weeks), medium term (22–30 weeks), and long term (over 48 weeks) using a random-effects model.

**Results:**

Twenty-nine studies with 2395 patients were included. Induction clinical remission and response was 61.15% and 57.48% for CD, and 40.07% and 64.72% for UC. Medium-term clinical remission and response was 60.72% and 64.32% for CD, and 43.51% and 64.87% for UC. Long-term clinical remission and response for CD was 45.83% and 44.55%, for UC was 56.14% and 61.35%. Endoscopic remission improved across induction, medium term, and long-term for CD (11.11%, 18.41% and 36.42%) and UC (43.19%, 54.27% and 66.72%). Within the longest follow up of 52 weeks, adverse event rate ranged widely from 6.67% to 61.54% for CD and 29.69% to 75.38% for UC. Chinese patients and both biologic-naïve and biologic-experienced patients showed similar favorable outcomes.

**Conclusion:**

VDZ demonstrated overall favourable effectiveness in Asian IBD patients, with a safety profile aligned with global data.

**Trial registration:**

The protocol was registered on National Institution for Health and Care Research (registration number CRD420251000408).

**Supplementary Information:**

The online version contains supplementary material available at 10.1186/s12876-026-04975-3.

## Background

Inflammatory bowel disease (IBD), comprising Crohn’s disease (CD) and ulcerative colitis (UC), is a chronic inflammatory disorder of the gastrointestinal tract [1, 2]. It manifests with symptoms like abdominal pain, diarrhoea, fatigue, and weight loss. Without effective management, it can lead to severe bowel damage and significant complications, severely impacting patients’ quality of life [3]. A study based on data from the Global Burden of Diseases, Injuries, and Risk Factors Study (GBD) 2017, reported that the age-standardized prevalence rate of IBD was 422.0 per 100,000 population in North America, and it varied between 104.5 and 161.5 in Europe. While, Asia showed a range of 15.3 to 134.6 per 100,000 population, with East Asia exhibiting the highest prevalence [4]. The clinical features of IBD in Asia differ from those in Western populations, including variations in disease progression, surgery rates, and the risk of colorectal cancer [5]. 

The treatment goals for IBD have evolved from symptom relief to achieving clinical and endoscopic remission, and preventing complications like colectomy and colorectal cancer [6–8]. Normalizing biomarkers is also recognized as an important short-term target closely linked to disease progression and treatment effectiveness [9]. Although advanced therapies like infliximab, the first anti-tumour necrosis factor (TNF) drug used in China, have improved disease control, approximately one-third of patients may not respond to anti-TNF agents initially, and about 40% may lose response during maintenance therapy due to the development of anti-drug antibodies (ADAs) or off-target effects [10, 11]. Thus, there is a pressing need for alternative treatment options.

Vedolizumab (VDZ), a humanized monoclonal antibody targeting the α4β7 integrin, reduces lymphocyte trafficking to the intestine and has demonstrated efficacy and safety in treating UC and CD in clinical trials [12–15]. In 2014, it was approved by the Food and Drug Administration (FDA) and European Medicines Agency (EMA) for the treatment of moderate-to-severe UC and CD [16]. Subsequently, around 2015–2020, it was approved in various Asian countries [17–19]. Additionally, growing real-world evidence indicates that VDZ is well-tolerated and effective in Asian patients with IBD [20]. However, a comprehensive systematic review or meta-analysis specifically evaluating the effectiveness and safety of VDZ in Asian populations in real world is lacking. Given the rising burden of IBD in Asia, this study aims to fill this critical gap by assessing the real-world effectiveness and safety of VDZ in Asian IBD patients.

## Methods

This review was performed in accordance with the Preferred Reporting Items for Systematic Review and Meta-Analysis (PRISMA 2020) extension statement [21]. The protocol was registered on National Institution for Health and Care Research (registration number CRD420251000408). As this paper is a systematic review, therefore Ethics Approval Statement and Informed Consent were not applicable.

### Data sources and literature search

An electronic database search of PubMed, EMBASE, the Cochrane Library, Web of Science, China Biology Medicine (CBM), China National Knowledge Infrastructure (CNKI), Wanfang, and VIP was conducted on 20th Dec 2023 using the following keywords: inflammatory bowel disease and vedolizumab. No restrictions were applied on language or publication status. Search strategy is provided in Supplementary 1.

### Study selection

We included studies involving patients diagnosed with IBD in Asia, specifically CD and UC. Eligible studies evaluated VDZ intravenous therapy, administered as 300 mg at weeks 0, 2, and 6, and subsequently every 8 weeks or every 4 weeks, provided the patient completed the induction phase. VDZ could be used alone or in combination with conventional medications including 5-Aminosalicylic acid (5-ASA), immunomodulators, and corticosteroids. Combination therapies with VDZ and other biological drugs or small molecules like infliximab (IFX), ustekinumab (UST), or adalimumab (ADA) were excluded. Eligible studies included cohort studies, post-marketing surveillance/safety studies (PMSS), pragmatic clinical trials, and case series. Only original manuscripts written in English or Chinese were included. Studies that designed as randomised controlled trials (RCTs) or case reports, or reported in other languages were excluded. Two reviewers independently screened articles for eligibility. Any disagreements were resolved through discussion.

Primary outcomes of interest were clinical remission rate, clinical response rate, and endoscopic remission rate. Secondary outcomes included corticosteroid-free clinical remission rate, endoscopic response rate, C-reactive protein (CRP) normalization rate (< 3 mg/L), faecal calprotectin (FC) normalization, treatment persistence rate, and safety variables such as overall adverse events (AE), serious adverse events (SAE), death, and the most common AEs. All outcomes were defined according to original text. Specific definitions from original text are shown in Supplementary 2. We considered studies with follow-up periods categorized as induction phase (6–14 weeks), medium term (22–30 weeks), and long-term (over 48 weeks).

### Data extraction

Two reviewers extracted the following data from each study into a pre-defined data extraction form: trial characteristics (i.e., author names, publication years, continent, country, study design, and sample size), participant characteristics (i.e., age, sex, disease, severity of disease, history of biologics, duration of disease ), intervention information (i.e., drug name, dosage, dose interval, treatment duration, and concomitant medication for disease), and outcome information (i.e., outcome name, definition, measurement timepoints). Any disagreements were resolved by discussion.

### Data synthesis and analysis

Meta-analyses were performed using R software (version 4.1.1) with a random-effects model. Separate analyses were performed for patients with CD and UC, focusing on predefined outcomes. For effectiveness variables, we extracted and calculated reported rates as proportions with 95% confidence intervals (95% CI) for each study, and then aggregated them using proportional meta-analysis. Subgroup analyses were performed for studies with available data, stratified by nation (China) and history of biologics (bio-naive vs. bio-exposure). Heterogeneity was assessed through clinical, methodological, and statistical dimensions, with *I²* values interpreted as indicating no, low (< 50%), moderate (50%-75%), or high (≥ 75%) heterogeneity [22]. Subgroup analyses were used to explore sources of substantial heterogeneity. Publication bias was evaluated using egger’s test for meta-analyses with more than ten studies. For safety variables, data from each study were summarized descriptively, presenting ranges of AE incidences. To evaluate the robustness of our pooled estimates and ensure the findings were not driven by variations in outcome definitions, we performed sensitivity analyses restricted to studies utilizing landmark trial standards. For CD, this involved analysing a subset of studies that strictly adhered to the gold-standard definitions of clinical response (a decrease in Crohn’s Disease Activity Index (CDAI) ≥ 100) and endoscopic remission (Simple Endoscopic Score for Crohn’s Disease (SES-CD) ≤ 4). Similarly, for UC, the analysis was limited to studies following the rigorous landmark criteria for clinical response (a decrease in Mayo score ≥ 3 and ≥ 30% from baseline with a rectal bleeding subscore decrease ≥ 1 or absolute score ≤ 1), clinical remission (Mayo score ≤ 2 with no subscore > 1), and endoscopic remission (Mayo endoscopic subscore of 0). Additionally, outcomes using alternative definitions identified in our literature search, such as a decrease in CDAI ≥ 70 and SES-CD ≤ 2 for CD, were analysed separately to assess their impact on the stability of the overall results.

### Quality assessment

We assessed controlled studies with multiple treatment groups using the Newcastle-Ottawa Scale (NOS), with a higher score (maximum score 9) indicates better quality [23]. For before-after (pre-post) studies without a control group, we applied the National Institutes of Health (NIH) quality assessment tool [24]. Any disagreements were resolved through discussion, with input from a third researcher if needed.

## Results

### Selection results

**Fig. 1:**
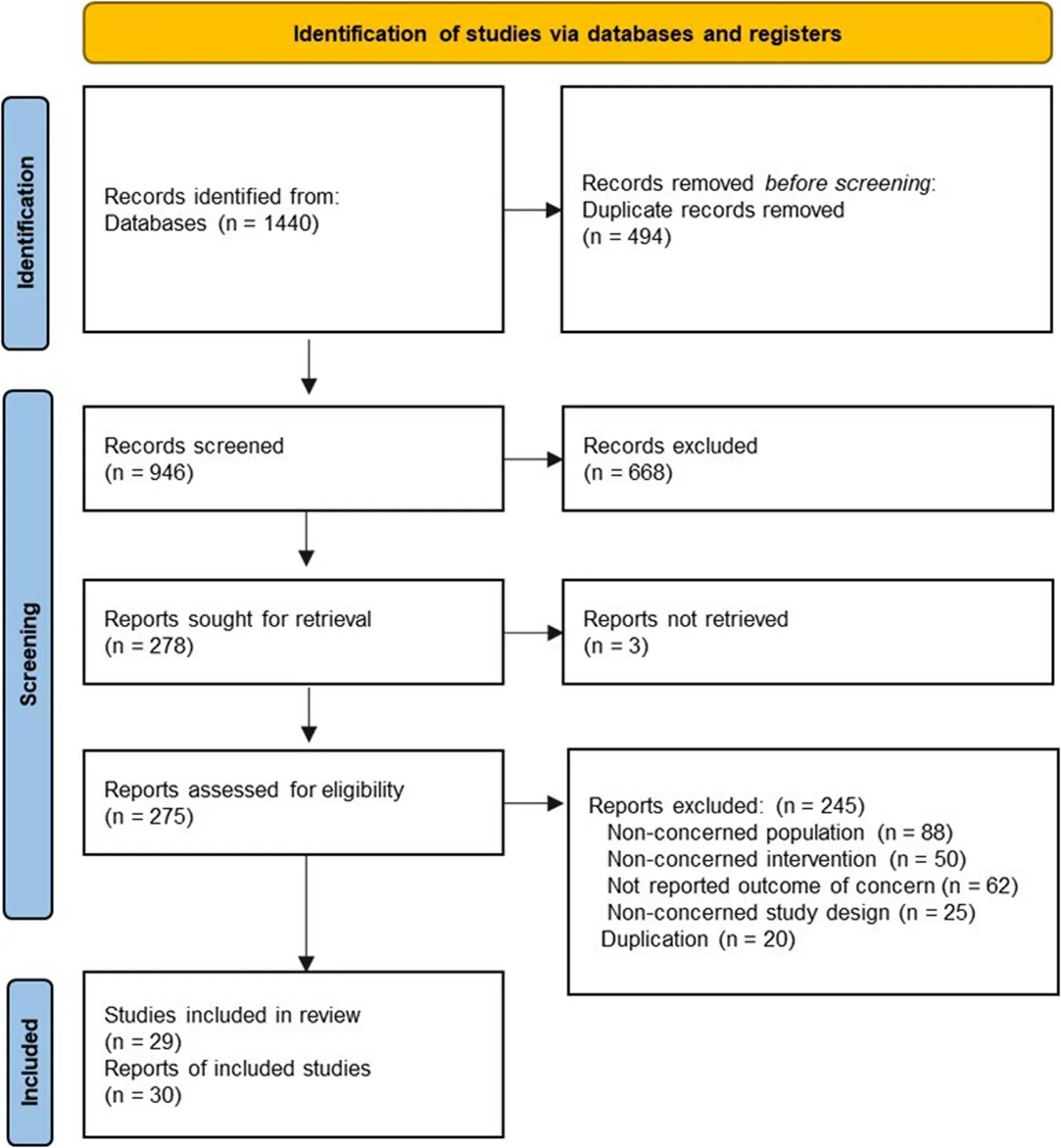
Flow diagram of the search process

Out of the 1440 retrieved records, 946 unique articles remained after deduplication. Following a review of titles and abstracts, 278 articles were selected for full-text screening. Three of these were inaccessible, leaving 275 full-text articles for evaluation. Ultimately, 30 articles reporting on 29 studies were included in the analysis (Fig. 1).

### Characteristics and quality of included studies

Of the 29 studies included (totalling 2395 patients), 15 studies [19, 25–39] reported data on CD patients, encompassing 827 individuals, while 25 studies [19, 25, 26, 28–31, 34, 35, 37–53] provided data on UC patients, with a total of 1568 individuals (Table S1). Among these, 20 studies specifically focused on Chinese patients (1472 patients), including 12 studies on CD (610 patients) and 15 studies on UC (862 patients).

Quality assessment was conducted using the NOS in six studies and the NIH quality assessment tool in 23 studies. Of the studies assessed by NOS, five achieved the maximum score of 9, while one study received a score of 7. For the NIH quality assessment, the majority of studies appropriately addressed key items. However, for two significant issues (sufficiency of sample size and loss to follow-up), all studies reported ‘cannot determine’, indicating that information regarding these specific criteria was either not provided or insufficient to make a conclusive assessment (Supplementary 3).

### Effectiveness across induction, medium- and long-term treatment periods

#### Induction phase

**Fig. 2:**
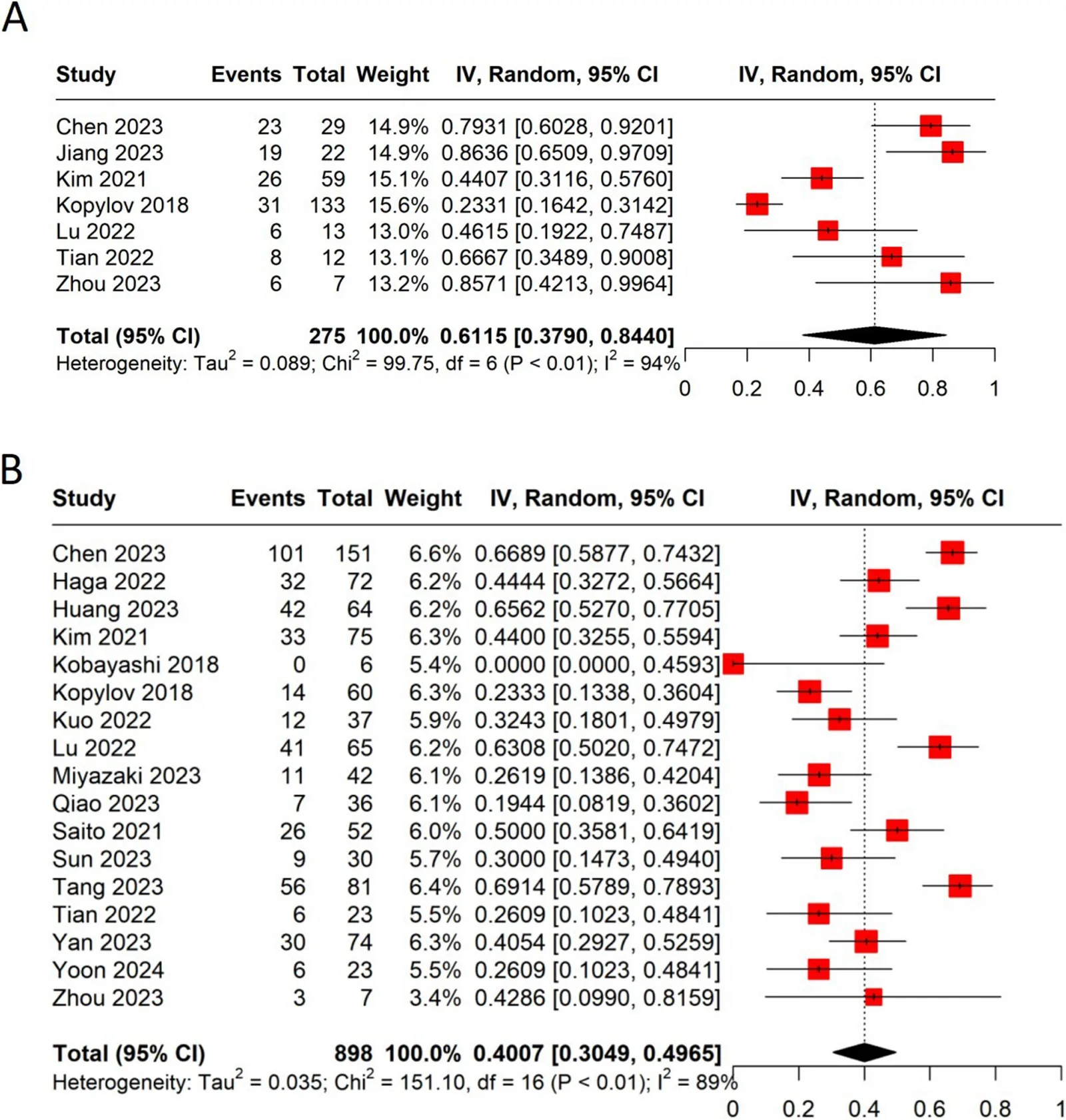
Forest plot of clinical remission during induction phase. **A** Crohn’s disease. **B** Ulcerative colitis

During the induction phase, clinical remission and response were achieved in 61.15% (95% CI 37.90% to 84.40%, 7 studies, 275 patients, Fig. 2A) and 57.48% (95% CI 32.93% to 82.03%, 7 studies, 267 patients, Fig. S1) of CD patients. For UC patients, the rates were 40.07% (95% CI 30.49% to 49.65%, 17 studies, 898 patients, Fig. 2B) and 64.72% (95% CI 54.67% to 74.76%, 16 studies, 830 patients, Fig. S2), respectively (Table 1). Corticosteroid-free clinical remission was observed in 35.59% (95% CI 23.55% to 49.13%) of CD patients, based on a single study with 59 patients, and in 47.12% (95% CI 32.73% to 61.50%, Fig. S3) of UC, based on 2 studies with 139 patients. Endoscopic outcomes for CD patients were less frequently reported, with an endoscopic remission rate of 11.11% (95% CI 5.08% to 22.60%, 4 studies, 54 patients, Fig. S4) and an endoscopic response rate of 41.37% (95% CI 23.45% to 59.30%, 2 studies, 29 patients, Fig. S5). For UC, endoscopic remission was 43.19% (95% CI 29.77% to 56.62%, 10 studies, 379 patients, Fig. S6) and the endoscopic response rate was 48.57% (95% CI 10.84% to 86.31%, 2 studies, 75 patients, Fig. S7). Additionally, CRP normalisation and treatment persistence rates for CD patients were 61.54% and 95.10%, respectively. For UC patients, the rates were 64.47% and 96.88%. The FC normalisation rate was 36.36% for UC patients (Table S2).

**Table 1:** Effectiveness in patients treated with vedolizumab in various follow-up periods

Outcomes	Crohn’s Disease				Ulcerative Colitis			
	Number of studies	Number of patients	Outcome data proportion [95% confidence interval]	Heterogeneity [I^2^]	Number of studies	Number of patients	Outcome data proportion [95% confidence interval]	Heterogeneity [I^2^]
Induction Phase								
Clinical remission rate	7	275	61.15% [37.90%, 84.40%]	94%	17	898	40.07% [30.49%, 49.65%]	89%
Clinical response rate	7	267	57.48% [32.93%, 82.03%]	95%	16	830	64.72% [54.67%, 74.76%]	91%
Corticosteroid-free clinical remission rate	1	59	35.59% [23.55%, 49.13%]	-	2	139	47.12% [32.73%, 61.50%]	67%
Endoscopic remission rate	4	54	11.11% [5.08%, 22.60%]	0%	10	379	43.19% [29.77%, 56.62%]	88%
Endoscopic response rate	2	29	41.37% [23.45%, 59.30%]	0%	2	75	48.57% [10.84%, 86.31%]	79%
Medium term								
Clinical remission rate	5	329	60.72% [46.47%, 74.97%]	85%	7	226	43.51% [25.55%, 61.46%]	88%
Clinical response rate	4	178	64.32% [45.69%, 82.94%]	86%	6	220	64.87% [46.28%, 83.46%]	91%
Corticosteroid-free clinical remission rate	2	203	36.72% [17.55%, 55.89%]	86%	-	-	-	-
Endoscopic remission rate	2	42	18.41% [1.95%, 34.87%]	46%	7	225	54.27% [45.77%, 62.78%]	39%
Endoscopic response rate	1	17	76.47% [50.10%, 93.19%]	-	2	76	42.08% [31.04%, 53.13%]	0%
Long term								
Clinical remission rate	6	429	45.83% [29.18%, 62.48%]	92%	9	648	56.14% [48.39%, 63.89%]	69%
Clinical response rate	2	189	44.55% [21.00%, 68.10%]	90%	6	558	61.35% [49.47%, 73.22%]	87%
Corticosteroid-free clinical remission rate	2	183	36.58% [29.61%, 43.56%]	0%	2	211	49.03% [20.26%, 77.80%]	94%
Endoscopic remission rate	3	87	36.42% [26.37%, 46.46%]	0%	6	228	66.72% [55.02%, 78.42%]	57%
Endoscopic response rate	-	-	-	-	-	-	-	-

Induction term: 6–14 weeks; medium term: 22–30 weeks; long term: over than 48 or 52 weeks

#### Medium term

**Fig. 3:**
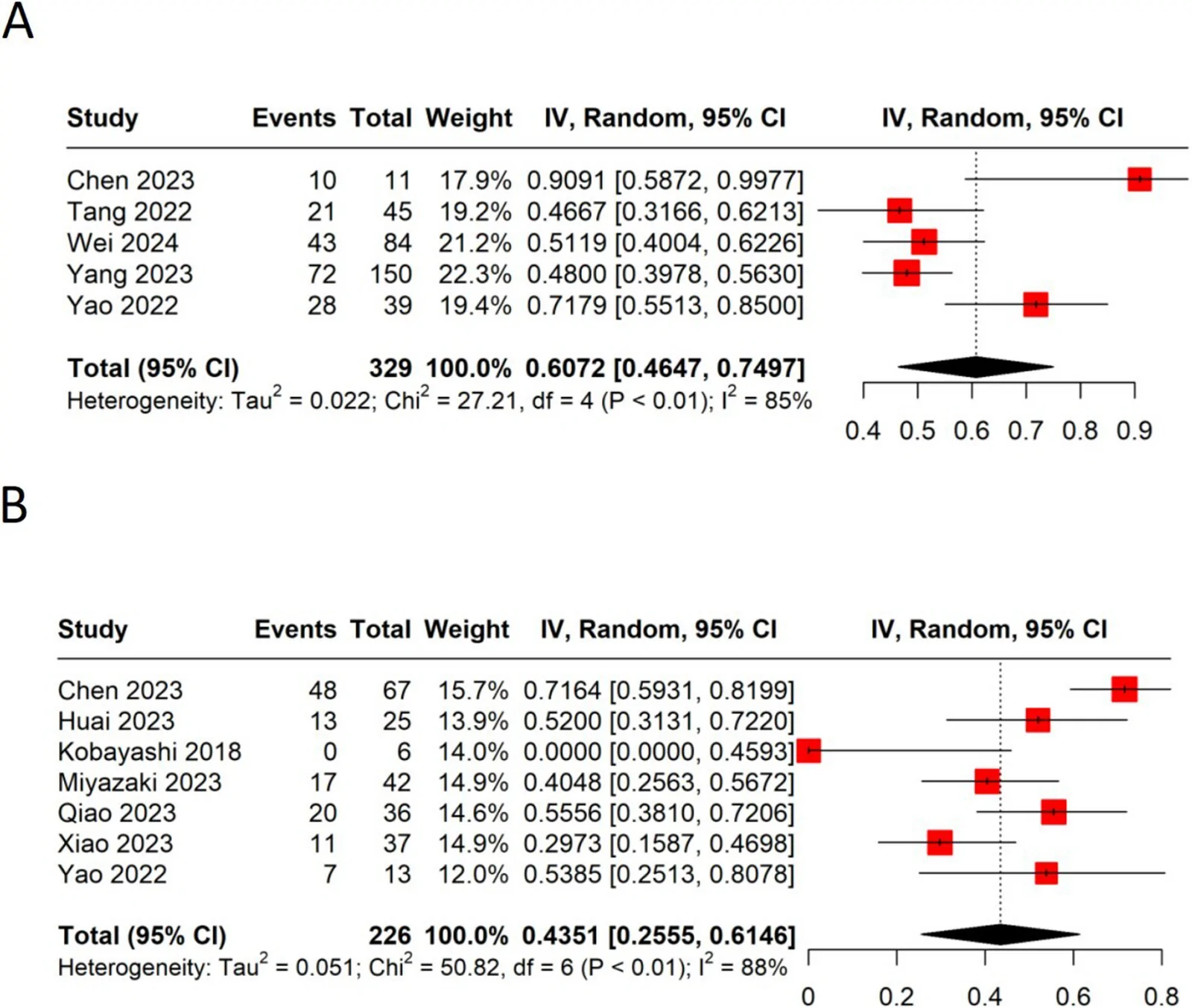
Forest plot of clinical remission in the medium term. **A** Crohn’s disease. **B** Ulcerative colitis

In the medium term, clinical remission rates were 60.72% (95% CI 46.47% to 74.97%, 5 studies, 329 patients, Fig. 3A) for CD and 43.51% (95% CI 25.55% to 61.46%,7 studies, 226 patients, Fig. 3B) for UC, while clinical response rates were 64.32% (95% CI 45.69% to 82.94%, 4 studies, 178 patients, Fig. S8) and 64.87% (95% CI 46.28% to 83.46%, 6 studies, 220 patients, Fig. S9), respectively. Corticosteroid-free clinical remission was 36.72% (95% CI 17.55% to 55.89%, Fig. S10) for CD, based on 2 studies with 203 patients. Endoscopic remission was 18.41% (95% CI 1.95% to 34.87%, Fig. S11) in CD as pooled from 2 studies with 42 patients, and 54.27% (95% CI 45.77% to 62.78%, Fig. S12) in UC as pooled from 7 studies with 225 patients. Endoscopic response reached 76.47% (95% CI 50.10% to 93.19%, 1 study, 17 patients) in CD and 42.08% (95% CI 31.04% to 53.13%, 2 studies, 76 patients, Fig. S13) in UC. Treatment persistence rates were 94.00% for CD and 94.40% for UC (Table S2).

#### Long term

**Fig. 4:**
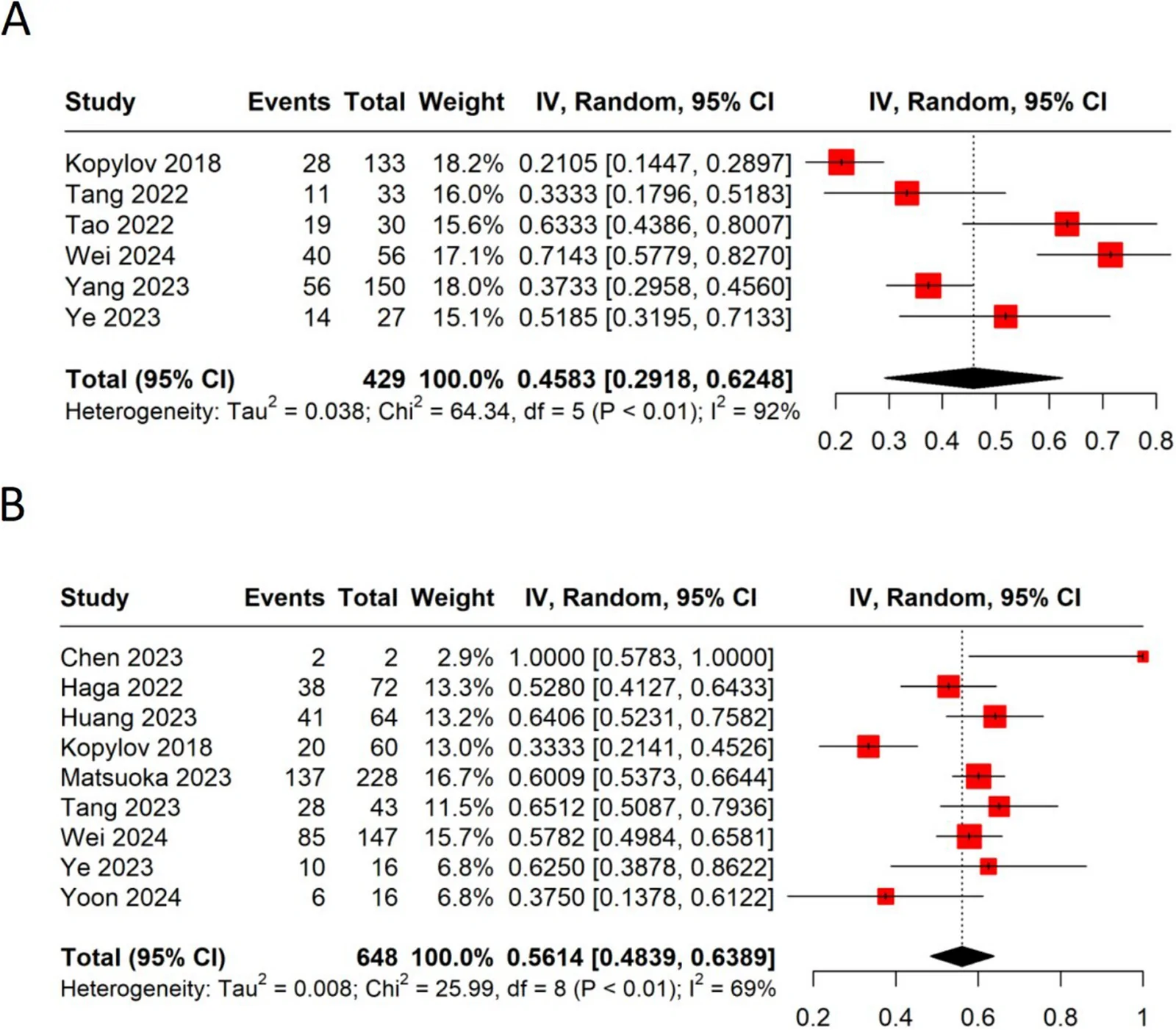
Forest plot of clinical remission in the long term. **A** Crohn’s disease. **B** Ulcerative colitis

In the long term, clinical remission rates were 45.83% (95% CI 29.18% to 62.48%, 6 studies, 429 patients, Fig. 4A) for CD and 56.14% (95% CI 48.39% to 63.89%, 9 studies, 648 patients, Fig. 4B) for UC. Clinical response rates reached 44.55% (95% CI 21.00% to 68.10%, 2 studies, 189 patients, Fig. S14) for CD and 61.35% (95% CI 49.47% to 73.22%, 6 studies, 558 patients, Fig. S15) for UC. Corticosteroid-free clinical remission was 36.58% (95% CI 29.61% to 43.56%, Fig. S16) for CD as pooled from 2 studies with 183 patients and 49.03% (95% CI 20.26% to 77.80%, Fig. S17) for UC as pooled from 2 studies with 211 patients. Endoscopic remission rates were 36.42% (95% CI 26.37% to 46.46%, 3 studies, 87 patients, Fig. S18) for CD and 66.72% (95% CI 55.02% to 78.42%, 6 studies, 228 patients, Fig. S19) for UC. Treatment persistence rates were 81.80% for CD and 93.55% for UC. Additionally, UC patients showed faecal calprotectin normalization and CRP normalisation rates of 33.33% and 63.64%, respectively (Table S2).

### Subgroup analysis

#### Clinical outcomes of Chinese population

The clinical outcomes for Chinese CD and UC patients across different time points are shown in Table 2. During the induction phase, the clinical remission rates were 75.58% (95% CI 62.93% to 88.22%) for CD and 46.46% (95% CI 34.10% to 58.83%) for UC, while clinical response rates were 71.31% (95% CI 39.48% to 100.00%) and 67.89% (95% CI 54.53% to 81.24%), respectively. Endoscopic remission rates were lower for CD at 11.11% (95% CI 5.08% to 22.60%) compared to 44.58% (95% CI 29.16% to 60.00%) for UC.

**Table 2:** Effectiveness in Chinese patients treated with vedolizumab in various follow-up periods

Outcomes	Crohn’s Disease				Ulcerative Colitis			
	Number of studies	Number of patients	Outcome data proportion [95% confidence interval]	Heterogeneity [I^2^]	Number of studies	Number of patients	Outcome data proportion [95% confidence interval]	Heterogeneity [I^2^]
Induction phase								
Clinical remission rate	5	83	75.58% [62.93%, 88.22%]	48%	10	568	46.46% [34.10%, 58.83%]	89%
Clinical response rate	4	71	71.31% [39.48%, 100.00%]	92%	10	566	67.89% [54.53%, 81.24%]	93%
Endoscopic remission rate	4	54	11.11% [5.08%, 22.60%]	0%	9	311	44.58% [29.16%, 60.00%]	89%
Medium term								
Clinical remission rate	5	329	60.72% [46.47%, 74.97%]	85%	4	141	60.95% [50.06%, 71.85%]	39%
Clinical response rate	4	178	64.32% [45.69%, 82.94%]	86%	4	141	65.72% [37.57%, 93.87%]	94%
Endoscopic remission rate	2	42	18.41% [1.95%, 34.87%]	46%	5	122	53.32% [39.86%, 66.77%]	56%
Long term								
Clinical remission rate	5	296	51.28% [35.27%, 67.30%]	86%	4	270	60.84% [55.03%, 66.65%]	0%
Clinical response rate	1	56	57.14% [43.22%, 70.29%]	-	3	254	73.50% [68.09%, 78.91%]	0%
Endoscopic remission rate	3	87	36.42% [26.37%, 46.46%]	0%	4	181	62.40% [55.03%,69.78%]	2%

Induction phase: 6-14 weeks; medium term: 22-30 weeks; long term: over than 48 or 52 weeks

In the medium term, clinical remission rates decreased to 60.72% (95% CI 46.47% to 74.97) for CD and 60.95% (95% CI 50.06% to 71.85%) for UC, with clinical response rates at 64.32% (95% CI 45.69% to 82.94%) for CD and 65.72% (95% CI 37.57% to 93.87%) for UC. Endoscopic remission rates remained relatively low for CD at 18.41% (95% CI 1.95% to 34.87%, 2 studies with 42 patients) but were higher for UC at 53.32% (95% CI 39.86% to 66.77%).

For long-term outcomes, clinical remission rates were 51.28% (95% CI 35.27% to 67.30%) for CD and 60.84% (95% CI 55.03% to 66.65%) for UC, while clinical response rates were 57.14% (95% CI 43.22% to 70.29%) and 73.50% (95% CI 68.09% to 78.91%), respectively. Endoscopic remission rates reached 36.42% (95% CI 26.37% to 46.46%) for CD and 62.40% (95% CI 55.03% to 69.78%) for UC.

#### Effectiveness stratified by history of biologics

Subgroup analysis based on history of biologic treatments revealed distinct patterns of effectiveness in both CD and UC patients across different time points (Table 3). During the induction phase, bio-exposed patients in CD achieved a clinical remission rate of 68.25% (95% CI 17.02% to 95.75%) and a clinical response rate of 46.07% (95% CI 27.15% to 66.18%), while bio-naïve patients had rates of 73.68% (95% CI 48.80% to 90.85%) and 36.84% (95% CI 16.29% to 61.64%), respectively. In UC, bio-exposed patients had a clinical remission rate of 45.22% (95% CI 36.24% to 54.52%) and a clinical response rate of 55.24% (95% CI 36.92% to 72.24%), while bio-naïve patients achieved rates of 54.71% (95% CI 40.09% to 68.57%) and 75.64% (95% CI 66.66% to 82.83%). Endoscopic remission rates in UC were 34.30% (95% CI 24.31% to 45.91%) for bio-exposed patients and 73.11% (95% CI 58.30% to 84.09%) for bio-naïve patients.

**Table 3:** Effectiveness in patients treated with vedolizumab in various follow-up periods according to history of biologics

Outcomes	Crohn’s Disease				Ulcerative Colitis			
	Number of studies	Number of patients	Outcome data proportion [95% confidence interval]	Heterogeneity [I^2^]	Number of studies	Number of patients	Outcome data proportion [95% confidence interval]	Heterogeneity [I^2^]
Induction phase								
Clinical remission rate								
Bio-exposure	2	69	68.25% [17.02%, 95.75%]	80%	6	148	45.22% [36.24%, 54.52%]	10%
Bio-naïve	1	19	73.68% [48.80%, 90.85%]	77%	5	303	54.71% [40.09%, 68.57%]	81%
Clinical response rate								
Bio-exposure	2	69	46.07% [27.15%, 66.18%]	40%	4	103	55.24% [36.92%, 72.24%]	49%
Bio-naïve	1	19	36.84% [16.29%, 61.64%]	-	3	225	75.64% [66.66%, 82.83%]	42%
Endoscopic remission rate								
Bio-exposure	-	-	-	-	3	73	34.30% [24.31%,45.91%]	0%
Bio-naïve	-	-	-	-	2	45	73.11% [58.30%, 84.09%]	0%
Medium term								
Clinical remission rate								
Bio-exposure	4	160	40.52% [33.12%, 48.37%]	0%	2	23	51.48% [31.49%,71.01%]	0%
Bio-naïve	4	130	59.33% [47.35%, 70.30%]	30%	1	63	71.43% [58.65%, 82.11%]	-
Clinical response rate								
Bio-exposure	3	59	50.66% [28.68%, 72.38%]	48%	1	4	75.00% [19.41%, 99.37%]	-
Bio-naïve	3	80	63.66% [52.56%, 73.47%]	0%	1	63	82.54% [70.90%, 90.95%]	-
Endoscopic remission rate								
Bio-exposure	1	10	10.00% [0.25%, 44.50%]	-	2	103	56.29% [46.58%, 65.54%]	0%
Bio-naïve	1	15	13.33% [1.66%, 40.46%]	-	1	17	76.47% [50.10%, 93.19%]	-
Long term								
Clinical remission rate								
Bio-exposure	2	112	43.84% [18.04%, 73.47%]	88%	1	29	51.72% [32.53%, 70.55%]	-
Bio-naïve	3	124	64.31% [43.25%, 80.99%]	77%	1	43	53.49% [37.65%, 68.82%]	-
Clinical response rate								
Bio-exposure	1	28	50.00% [30.65%, 69.35%]	-	-	-	-	-
Bio-naïve	1	28	64.29% [44.07%, 81.36%]	-	-	-	-	-
Endoscopic remission rate								
Bio-exposure	1	13	46.15% [19.22%, 74.87%]	-	2	25	75.00% [47.51%, 90.86%]	36%
Bio-naïve	2	47	31.03% [11.63%, 60.61%]	67%	2	42	69.93% [47.46%, 85.68%]	42%

Induction phase: 6–14 weeks; medium term: 22–30 weeks; long term: over than 48 or 52 weeks

In the medium term, CD bio-exposed patients achieved a clinical remission rate of 40.52% (95% CI 33.12% to 48.37%) and a clinical response rate of 50.66% (95% CI 28.68% to 72.38%). Endoscopic remission was observed in 10.00% (95% CI 0.25% to 44.50%) of patients. Bio-naïve patients had a clinical remission rate of 59.33% (95% CI 47.35% to 70.30%) and a clinical response rate of 63.66% (95% CI 52.56% to 73.47%). Endoscopic remission was observed in 13.33% (95% CI 1.66% to 40.46%) of bio-naïve patients. In UC, bio-exposed patients showed a clinical remission rate of 51.48% (95% CI 31.49% to 71.01%) and a clinical response rate of 75.00% (95% CI 19.41% to 99.37%), with an endoscopic remission rate of 56.29% (95% CI 46.58% to 65.54%). Bio-naïve patients achieved a clinical remission rate of 71.43% (95% CI 58.65% to 82.11%) and a clinical response rate of 82.54% (95% CI 70.90% to 90.95%), with an endoscopic remission rate of 76.47% (95% CI 50.10% to 93.19%).

For long-term outcomes, CD bio-exposed patients showed a clinical remission rate of 43.84% (95% CI 18.04% to 73.47%), a clinical response rate of 50.00% (95% CI 30.65% to 69.35%), and an endoscopic remission rate of 46.15% (95% CI 19.22% to 74.87%). Bio-naïve patients had a clinical remission rate of 64.31% (95% CI 43.25% to 80.99%), a clinical response rate of 64.29% (95% CI 44.07% to 81.36%), and an endoscopic remission rate of 31.03% (95% CI 11.63% to 60.61%). In UC, bio-exposed patients achieved a clinical remission rate of 51.72% (95% CI 32.53% to 70.55%) and an endoscopic remission rate of 75.00% (95% CI 47.51% to 90.86%). Bio-naïve patients had a clinical remission rate of 53.49% (95% CI 37.65% to 68.82%) and an endoscopic remission rate of 69.93% (95% CI 47.46% to 85.68%).

### Sensitivity and robustness analyses

Overall, the results were consistent with the primary analysis, though some variations were noted in specific endpoints (Table S3). In the induction phase, clinical remission rates for CD and UC remained stable (CD: 70.04% vs. 61.15%; UC: 39.56% vs. 40.07%). Notably, the CD clinical response rate was 67.13% using CDAI ≥ 100 threshold, and 95.45% using CDAI ≥ 70 threshold. Furthermore, applying the strictest endoscopic criteria led to lower reported remission rates in the induction phase, particularly for UC (27.98% vs. 43.19% in the primary analysis). For medium- and long-term phases, the sensitivity analyses confirmed the robustness of the primary estimates. Clinical remission and response rates generally aligned with original findings. Although endoscopic remission rates exhibited slight fluctuations (e.g., UC long-term: 50.61% vs. 66.72%), the longitudinal trend of increasing mucosal healing remained consistent. These findings suggest that while specific definitions may impact absolute percentages, the overall therapeutic signals and the durability of the response remain reliable.

### Safety for CD and UC patients

AE rates ranged from 6.67% to 61.54% in CD patients, and 29.69% to 75.38% in UC patients. SAEs in CD patients were infrequently reported, with one study noting an SAE rate of 0.00% (Table S4). For UC patients, SAEs were reported in three studies, with rates from 0.00% to 1.56%. Discontinuation of treatment varied from 1.25% to 6.45% in CD patients, and 0.00% to 5.13% in UC patients (Table S5). Common AEs included arthralgia, rash, allergic reaction and Clostridium difficile infection (CDI) (Table S4-5).

No distinct safety concerns were identified in Chinese CD (Table S6) and UC patients (Table S7) beyond those observed in the Asian population.

### Publication bias

Egger’s test was conducted for clinical remission rate, clinical response rate, and endoscopic remission rate. The results showed no significant for most of the outcomes, but significant for clinical remission rate for UC patients in the induction phase (*p* = 0.0143).

## Discussion

To the best of our knowledge, this systematic review and meta-analysis is the first to synthesized published evidence evaluating the real-world effectiveness and safety profile of VDZ in the Asian IBD population, reaffirming its therapeutic utility in managing CD and UC in this demographic. It provides a comprehensive exploration of subgroup results specific to the Chinese population, with particular attention to the influence of prior biologic exposure. The unique focus on Asian patients provides novel insights into the practical application of VDZ within this specific cohort. By categorising effectiveness across distinct follow-up intervals—induction (6–14 weeks), medium term (22–30 weeks), and long term (over 48 weeks)—our findings offer a granular analysis of VDZ’s temporal effectiveness trends.

Among UC patients, clinical remission rates for Asian patients were comparable to global data [54], with 40.07% achieving clinical remission in induction, 43.51% in the medium term, and 56.14% in the long term. These rates align with global averages of approximately 40% during induction and 45% during the maintenance phase [54]. Moreover, we found Asian patients might benefit more from VDZ treatment in terms of long-term outcomes. But these results might be influenced by baseline patients characteristics, such as milder disease activity, lower prevalence of prior biologic exposure and should be interpreted with caution. Subgroup analysis indicated that the clinical remission and response rates were similar between biologic-naïve patients and those with prior biologic exposure, though the rates were numerically higher in the biologic-naïve group. This observation is consistent with prior research, including a post-hoc analysis of GEMINI 1, which demonstrated heightened efficacy of VDZ in TNF-naïve patients compared to those with prior anti-TNF exposure [55]. The relatively low prevalence of biologic use in Asia prior to VDZ approval may have contributed to the higher proportion of biologic-naïve patients in these studies, thereby influencing the improved overall outcomes [56]. 

In the context of a treat-to-target approach in IBD management, achieving mucosal healing, which is assessing via endoscopy, is a critical therapeutic goal. The GEMINI 1 study provided valuable insights into the efficacy of VDZ in treating both global and Asian patients with UC [15, 17]. At week 52, the study reported mucosal healing rates of 51.6% for VDZ administered every 8 weeks and 56.0% for VDZ administered every 4 weeks in the global UC population [15]. In the Asian subgroup, the corresponding rates were 45.5% and 47.4%, respectively [17]. Our meta-analysis of Asian patients revealed similar and, in some cases, even higher rates of endoscopic remission. Over the long term, the endoscopic remission rate for UC was 66.72%, with a complete endoscopic remission rate of 50.61%. These long-term findings further underscore the durability of VDZ’s effectiveness in Asian patients, aligning closely with the results of the GEMINI 1 study.

For CD patients in Asia, clinical remission rates were 61.15% during induction, 60.72% in the medium term, and 45.83% in the long term. These rates appear higher than the global meta-analysis rates of 36% during induction and 39% during maintenance (26–52 weeks) [54]. In the GEMINI 2 Asian subgroup, the remission rate was 14.7% during induction and 41.7% (every 4 weeks VDZ) and 36.4% (every 8 weeks VDZ) in the maintenance phase for CD patients [57]. However, this comparison should be interpreted with caution given the small sample size of the Asian subgroup in GEMINI 2. The favorable outcomes observed in our analysis may be influenced by real treatment patterns, such as earlier use of VDZ and a higher proportion of biologic-naïve patients in Asia. However, further evidence, including comparative data on the proportion of biologic-naïve patients and disease duration between Asian and Western populations, is needed to substantiate these observations [58]. Moreover, we observed higher clinical remission rates than clinical response rates in CD patients, a finding that appears counterintuitive relative to routine clinical experience. This discrepancy may be attributed to the evolving acceptance of early biologic initiation in clinical practice, which results in some patients with relatively mild disease severity were enrolled and makes clinical remission more readily attainable than clinical response under standard assessment criteria. Notably, revised definitions of clinical response have been proposed for real-world settings. For instance, Onali 2022 classified patients who achieve either remission or response as clinical responders [59]. Nevertheless, our analytical approach was based strictly on the outcome definitions and data as reported in the original included studies.

In terms of endoscopic remission, the VERSIFY study showed an increase with VDZ from 11.9% at week 26 to 17.9% at week 52, with numerically higher rates in biologic-naïve patients [60]. Similarly, the LOVE-CD study reported higher remission rates in early versus late CD (31.4% vs. 8.6%) [61]. In our analysis, VDZ was associated with a trend toward endoscopic improvement in Asian patients with CD, with endoscopic remission increasing from 11.1% during induction to 36.4% in the long term. Bio-naïve patients appeared to have higher endoscopic remission rates than biologic-exposed patients, particularly in the medium term. However, given the small sample sizes in these subgroups, these findings should be interpreted with caution.

Notably, substantial heterogeneity was observed across pooled outcomes (*I²* frequently > 80–90%), likely reflecting underlying clinical and methodological variability among included real-world studies. Clinically, differences in baseline disease severity, Montreal classification, and concomitant therapies (e.g., immunomodulators and corticosteroids) may have significantly influenced treatment response. In contrast, as all included studies were cohort in design, study design is unlikely to be a major source of heterogeneity, and variation in follow-up duration, which was explored through subgroup analyses, did not fully account for the observed variability. Additional contributors may include incomplete reporting of disease phenotypes, limiting more refined analyses. Given the descriptive nature of single-arm meta-analysis, the pooled estimates should be interpreted as a reflection of the range of observed outcomes rather than precise effect sizes. Collectively, these factors underscore the complexity of synthesizing real-world evidence and highlight the need for more standardized reporting and larger studies to better characterize sources of heterogeneity.

Our findings support a favourable safety profile for VDZ in Asian populations, consistent with published data and without any new safety signals. For CD, the incidence of AEs ranged from 6.67% to 61.54%, and for UC, it was 29.69% to 75.38 with SAEs notably low at 0.00% to 1.56% in UC and none reported in CD. These rates are significantly lower than those reported in the GEMINI studies where AE and SAE rates during the induction and maintenance phases were 57% and 7%, and 87% and 24% for CD, and 45% and 3%, and 80% and 12% for UC, respectively [15]. The most common AEs observed—infusion reactions, infections, and gastrointestinal discomfort—align with VDZ’s established safety profile. These results suggest that VDZ offers a safety advantage, particularly for Asian, including Chinese, populations.

### Strengths and limitations

This study is among the first meta-analyses to focus exclusively on Asian cohorts, offering valuable insights into the therapeutic potential of VDZ in this population. The large sample size, broad study inclusion, comprehensive assessment of clinical and endoscopic outcomes, and detailed subgroup analyses of prior biologic exposure further enhance the strengths of this review. Moreover, by including a large number of observational studies, we have drawn conclusions regarding the real-world effectiveness and safety, which can offer guidance to clinical practice that is comparably valid. Nevertheless, some limitations should be noted. The predominance of Chinese studies may limit the applicability of findings to other Asian sub-populations. The observed substantial statistical and clinical heterogeneity may limit the interpretability of the pooled estimates. One potential source of heterogeneity is the variability in the definitions of effectiveness outcomes across the included studies, as detailed in Supplementary Table 2. Differences in outcome assessment criteria and evaluation time points may have affected the comparability of results and contributed to between-study heterogeneity. To address this, we conducted sensitivity analyses restricted to landmark trial definitions, which confirmed that our primary findings are largely robust. While therapeutic trends remained consistent, absolute rates expectedly fluctuated based on the stringency of the criteria. For instance, the decline in UC endoscopic remission rates under the strictest landmark criteria (from 43.19% to 27.98%) underscores the higher bar for achieving complete mucosal healing versus mere improvement. These variations highlight the importance of standardized endpoints but ultimately reinforce the reliability of VDZ’s effectiveness across different clinical thresholds.

In addition, the risk-of-bias assessment indicated that most included studies were of moderate quality based on the NIH tool, with common limitations including relatively small sample sizes and loss to follow-up, which may have affected the reliability and generalizability of the pooled estimates. Furthermore, we found that studies reporting endoscopic outcomes in patients with CD are limited. To build on these findings, future research should prioritise large-scale, prospective studies with extended follow-up periods to validate these real-world findings and further explore the factors contributing to the differential effectiveness of VDZ across Asian populations. It should be noted that Egger’s test indicated significant publication bias for clinical remission during the induction phase in patients with UC. This suggests that studies reporting favourable outcomes may be more likely to be published, which could lead to an overestimation of the pooled effectiveness of VDZ in this setting. Therefore, the observed remission rates should be interpreted with caution. Moreover, head-to-head trials comparing VDZ with other biologics or small-molecule therapies in Asian patients would provide more robust evidence to guide clinical decision-making.

## Conclusion

Our review indicates that VDZ is a safe and effective treatment for Asian patients with IBD, with particularly favourable efficacy observed in UC. However, these findings should be interpreted with caution given the heterogeneity of the included studies and the predominantly observational nature of the evidence. Future studies should validate these findings and explore effectiveness variations across different Asian populations.

## Supplementary Information

Supplementary Material 1.

## Data Availability

The datasets supporting the conclusions of this article are included within the article and its additional files.

## Abbreviations

ADA: Adalimumab
AE: Adverse Events
CBM: China Biology Medicine
CD: Crohn’s Disease
CNKI: China National Knowledge Infrastructure
CRP: C-reactive Protein
FC: Faecal Calprotectin
IBD: Inflammatory Bowel Disease
NIH: National Institutes of Health
NOS: Newcastle-Ottawa Scale
SAE: Serious Adverse Events
TNF: Tumour Necrosis Factor
UC: Ulcerative Colitis
VDZ: Vedolizumab
