# Supplementary material for: Effectiveness and safety of vedolizumab in real-world studies in Asian patients with inflammatory bowel disease: a systematic review and meta-analysis
Source: BMC Gastroenterol. 2026 Jun 12;26:525. doi: 10.1186/s12876-026-04975-3 (PMC13505098; doi:10.1186/s12876-026-04975-3)
Supplement: Supplementary file 1 [file 12876_2026_4975_MOESM1_ESM.docx]

***Content***

**[Supplementary 1 Search strategy](#_Toc229733200)**	[2](#_Toc229733200)

**[Supplementary 2 Specific definitions from original studies](#_Toc229733201)**	[7](#_Toc229733201)

**[Supplementary 3 Quality assessment results](#_Toc229733202)**	[9](#_Toc229733202)

**[Risks of bias assessment (Crohn’s Disease -](#_Toc229733203)** **[Newcastle-Ottawa Scale)](#_Toc229733203)**	[9](#_Toc229733203)

**[Risks of bias assessment (Crohn’s Disease-](#_Toc229733204)** **[National Institutes of Health)](#_Toc229733204)**	[10](#_Toc229733204)

**[Risks of bias assessment (Ulcerative Colitis-](#_Toc229733205)** **[Newcastle-Ottawa Scale)](#_Toc229733205)**	[12](#_Toc229733205)

**[Risks of bias assessment (Ulcerative Colitis -](#_Toc229733206)** **[National Institutes of Health)](#_Toc229733206)**	[13](#_Toc229733206)

**[Table S1 Baseline characteristics of included studies](#_Toc229733207)**	[15](#_Toc229733207)

**[Table S2 Results for Asian population](#_Toc229733208)**	[21](#_Toc229733208)

**[Table S3 Effectiveness in patients treated with vedolizumab in various follow-up periods-sensitivity analysis](#_Toc229733209)**	[22](#_Toc229733209)

**[Table S4 Summary of safety outcomes of Crohn’s Disease in Asian patients receiving VDZ](#_Toc229733210)**	[23](#_Toc229733210)

**[Table S5 Summary of safety outcomes of Ulcerative Colitis in Asian patients receiving VDZ](#_Toc229733211)**	[24](#_Toc229733211)

**[Table S6 Summary of safety outcomes of Crohn’s Disease in Chinese patients receiving VDZ](#_Toc229733212)**	[27](#_Toc229733212)

**[Table S7 Summary of safety outcomes of Ulcerative Colitis in Chinese patients receiving VDZ](#_Toc229733213)**	[28](#_Toc229733213)

**[Forest plots for efficacy in Asian patients with Crohn’s Disease and Ulcerative Colitis during induction phase](#_Toc229733214)**	[30](#_Toc229733214)

**[Forest plots for efficacy in Asian patients with Crohn’s Disease and Ulcerative Colitis in medium term](#_Toc229733215)**	[33](#_Toc229733215)

**[Forest plots for efficacy in Asian patients with Crohn’s Disease and Ulcerative Colitis in long term](#_Toc229733216)**	[35](#_Toc229733216)

**Supplementary 1 Search strategy**

**PubMed**

#1. "Inflammatory Bowel Diseases"[Mesh] OR Inflammatory Bowel Diseas*[tw] OR IBD[tw] OR "Crohn-s Enteriti*"[tw] OR Crohn Enteriti*[tw] OR Crohns Enteriti*[tw] OR "Crohn-s Diseas*"[tw] OR Crohn Diseas*[tw] OR Crohns Diseas*[tw] OR Regional Enteriti*[tw] OR Granulomatous Enteriti*[tw] OR Ileocoliti*[tw] OR Granulomatous Coliti*[tw] OR Terminal Ileiti*[tw] OR Regional Ileiti*[tw] OR Idiopathic Proctocolit*[tw] OR Ulcerative Coliti*[tw] OR Colitis Gravis*[tw]
#2. "vedolizumab" [Supplementary Concept] OR vedolizumab[tw] OR Entyvio[tw] OR "mln 02"[tw] OR kynteles[tw] OR "mln 0002"[tw] OR MLN002[tw] OR "MLN-002"[tw] OR MLN02[tw] OR "MLN-02"[tw] OR "LDP-02"[tw] OR "anti-alpha4beta7"[tw] OR "alpha4beta7 inhibit*"[tw] OR "alpha4beta7 antibod*"[tw] OR "Anti-α4β7"[tw] OR "α4β7 inhibit*"[tw] OR "α4β7 antibod*"[tw]
#3. #1 AND #2
#4. "Asia"[Mesh] OR Asia[tw] OR Asian[tw] OR Asians[tw] OR Asiatic[tw] OR China OR Chinese OR Taiwan OR Taiwanese OR "Hong kong" OR Hongkong OR Macau OR Macao OR Beijing OR Shanghai OR Tianjin OR Chongqing OR "Inner Mongolia" OR Tibet OR Guangxi OR Sinkiang OR Ningxia OR Xinjiang OR Hebei OR Shanxi OR Liaoning OR Jilin OR Heilongjiang OR Jiangsu OR Zhejiang OR Anhui OR Fujian OR Jiangxi OR Shandong OR Henan OR Hubei OR Hunan OR Guangdong OR Hainan OR Sichuan OR Guizhou OR Yunnan OR Shaanxi OR Gansu OR Qinghai OR Japan OR Japanese OR Korea OR Korean OR Mongolia OR Mongolian OR Afghanistan OR Afghan OR UAE OR "United Arab Emirates" OR Arab OR Arabic OR Oman OR omanis OR omani OR Azerbaijan OR Azerbaijani OR Pakistan OR Pakistani OR Palestine OR Palestinian OR Bahrain OR bahrainese OR bahraini OR Bhutan OR bhutanese OR "East Timor" OR "Timor-Leste" OR Timorese OR Philippines OR Philippine OR philippinese OR Georgia OR Georgian OR Kazakhstan OR Kyrgyzstan OR Cambodia OR Cambodian OR Qatar OR Qatari OR Kuwait OR kuwaitis OR Laos OR Lao OR Lebanon OR Lebanese OR Maldives OR maldivian* OR Malaysia OR Malaysian OR Bangladesh OR Bangladeshi OR Burma OR Myanmar OR Burmese OR Nepal OR nepalese OR Saudi Arabia OR Saudi Arabian OR Sri Lanka OR Sri Lankan OR Tajikistan OR Tajik OR Thailand OR Thai OR Turkey OR Turkish OR Turkmenistan OR Brunei OR Bruneian* OR Uzbekistan OR Singapore OR Singaporean OR Syria OR syrian* OR Armenia OR Armenian* OR Yemen OR Yemenese OR Iraq OR Iraqi OR Iran OR Iranian* OR Israel OR Israeli OR India OR Indian OR Indonesia OR indonesian* OR Jordan OR Jordanese OR Vietnam OR Vietnamese OR "Viet Nam"
#5. #3 AND #4
#6. "Review Literature as Topic"[Mesh] OR "Review"[pt] OR Review[ti] OR "Case Reports"[pt] OR "Meta-Analysis"[pt] OR "Meta-Analysis as Topic"[Mesh] OR "Systematic Review"[pt] OR "Systematic Reviews as Topic"[Mesh] OR "systematic"[Filter] OR "Case Report"[ti] OR "A Case"[ti] OR "Meta-Analysis"[ti] OR Metaanalys*[ti] OR ("Animals"[Mesh] NOT ("Humans"[Mesh] AND "Animals"[Mesh]))
#7. #5 NOT #6

**EMBASE**

#1. 'inflammatory bowel disease'/exp OR ("Inflammatory Bowel Diseas*" OR IBD OR (("Crohn-s" OR Crohn OR Crohns OR Regional* OR Granulomatous) NEAR/3 (Enteriti* OR Diseas*)) OR Ileocoliti* OR ((Granulomatous OR Terminal OR Regional OR Idiopathic OR Ulcerati* OR ulcerosa OR mucosal OR ulcerous OR Gravis*) NEAR/3 (Coliti* OR Ileiti* OR Proctocolit*))):ab,ti,kw
#2. 'vedolizumab'/exp OR (vedolizumab OR Entyvio OR "mln 02" OR kynteles OR "mln 0002" OR MLN002 OR "MLN-002" OR MLN02 OR "MLN-02" OR "LDP-02" OR "anti-alpha4beta7" OR "alpha4beta7 inhibit*" OR "alpha4beta7 antibod*" OR "Anti-α4β7" OR "α4β7 inhibit*" OR "α4β7 antibod*"):ab,ti,kw
#3. #1 AND #2
#4. 'Asia'/exp OR (Asia OR Asian OR Asians OR Asiatic OR China OR Chinese OR Taiwan OR Taiwanese OR "Hong kong" OR Hongkong OR Macau OR Macao OR Beijing OR Shanghai OR Tianjin OR Chongqing OR "Inner Mongolia" OR Tibet OR Guangxi OR Sinkiang OR Ningxia OR Xinjiang OR Hebei OR Shanxi OR Liaoning OR Jilin OR Heilongjiang OR Jiangsu OR Zhejiang OR Anhui OR Fujian OR Jiangxi OR Shandong OR Henan OR Hubei OR Hunan OR Guangdong OR Hainan OR Sichuan OR Guizhou OR Yunnan OR Shaanxi OR Gansu OR Qinghai OR Japan OR Japanese OR Korea OR Korean OR Mongolia OR Mongolian OR Afghanistan OR Afghan OR UAE OR "United Arab Emirates" OR Arab OR Arabic OR Oman OR omanis OR omani OR Azerbaijan OR Azerbaijani OR Pakistan OR Pakistani OR Palestine OR Palestinian OR Bahrain OR bahrainese OR bahraini OR Bhutan OR bhutanese OR "East Timor" OR "Timor-Leste" OR Timorese OR Philippines OR Philippine OR philippinese OR Georgia OR Georgian OR Kazakhstan OR Kyrgyzstan OR Cambodia OR Cambodian OR Qatar OR Qatari OR Kuwait OR kuwaitis OR Laos OR Lao OR Lebanon OR Lebanese OR Maldives OR maldivian* OR Malaysia OR Malaysian OR Bangladesh OR Bangladeshi OR Burma OR Myanmar OR Burmese OR Nepal OR nepalese OR Saudi Arabia OR Saudi Arabian OR Sri Lanka OR Sri Lankan OR Tajikistan OR Tajik OR Thailand OR Thai OR Turkey OR Turkish OR Turkmenistan OR Brunei OR Bruneian* OR Uzbekistan OR Singapore OR Singaporean OR Syria OR syrian* OR Armenia OR Armenian* OR Yemen OR Yemenese OR Iraq OR Iraqi OR Iran OR Iranian* OR Israel OR Israeli OR India OR Indian OR Indonesia OR indonesian* OR Jordan OR Jordanese OR Vietnam OR Vietnamese OR "Viet Nam"):ti,ab,ad,ff
#5. #3 AND #4
#6. 'case report'/exp OR 'review'/exp OR 'review'/it OR 'meta analysis'/exp OR 'meta analysis (topic)'/exp OR 'systematic review'/exp OR 'systematic review (topic)'/exp OR (review OR "case report" OR "a case" OR 'Meta Analysis*' OR Metaanalys*):ti OR (('nonhuman'/exp OR 'animal'/exp) NOT 'human'/exp)
#7. #5 NOT #6

**WOS**

#1. TS=("Inflammatory Bowel Diseas*" OR IBD OR (("Crohn-s" OR Crohn OR Crohns OR Regional* OR Granulomatous) NEAR/3 (Enteriti* OR Diseas*)) OR Ileocoliti* OR ((Granulomatous OR Terminal OR Regional OR Idiopathic OR Ulcerati* OR ulcerosa OR mucosal OR ulcerous OR Gravis*) NEAR/3 (Coliti* OR Ileiti* OR Proctocolit*)))
#2. TS=(vedolizumab OR Entyvio OR "mln 02" OR kynteles OR "mln 0002" OR MLN002 OR "MLN-002" OR MLN02 OR "MLN-02" OR "LDP-02" OR "anti-alpha4beta7" OR "alpha4beta7 inhibit*" OR "alpha4beta7 antibod*" OR "Anti-α4β7" OR "α4β7 inhibit*" OR "α4β7 antibod*")
#3. #1 AND #2
#4. TS=(Asia OR Asian OR Asians OR Asiatic OR China OR Chinese OR Taiwan OR Taiwanese OR "Hong kong" OR Hongkong OR Macau OR Macao OR Beijing OR Shanghai OR Tianjin OR Chongqing OR "Inner Mongolia" OR Tibet OR Guangxi OR Sinkiang OR Ningxia OR Xinjiang OR Hebei OR Shanxi OR Liaoning OR Jilin OR Heilongjiang OR Jiangsu OR Zhejiang OR Anhui OR Fujian OR Jiangxi OR Shandong OR Henan OR Hubei OR Hunan OR Guangdong OR Hainan OR Sichuan OR Guizhou OR Yunnan OR Shaanxi OR Gansu OR Qinghai OR Japan OR Japanese OR Korea OR Korean OR Mongolia OR Mongolian OR Afghanistan OR Afghan OR UAE OR "United Arab Emirates" OR Arab OR Arabic OR Oman OR omanis OR omani OR Azerbaijan OR Azerbaijani OR Pakistan OR Pakistani OR Palestine OR Palestinian OR Bahrain OR bahrainese OR bahraini OR Bhutan OR bhutanese OR "East Timor" OR "Timor-Leste" OR Timorese OR Philippines OR Philippine OR philippinese OR Georgia OR Georgian OR Kazakhstan OR Kyrgyzstan OR Cambodia OR Cambodian OR Qatar OR Qatari OR Kuwait OR kuwaitis OR Laos OR Lao OR Lebanon OR Lebanese OR Maldives OR maldivian* OR Malaysia OR Malaysian OR Bangladesh OR Bangladeshi OR Burma OR Myanmar OR Burmese OR Nepal OR nepalese OR Saudi Arabia OR Saudi Arabian OR Sri Lanka OR Sri Lankan OR Tajikistan OR Tajik OR Thailand OR Thai OR Turkey OR Turkish OR Turkmenistan OR Brunei OR Bruneian* OR Uzbekistan OR Singapore OR Singaporean OR Syria OR syrian* OR Armenia OR Armenian* OR Yemen OR Yemenese OR Iraq OR Iraqi OR Iran OR Iranian* OR Israel OR Israeli OR India OR Indian OR Indonesia OR indonesian* OR Jordan OR Jordanese OR Vietnam OR Vietnamese OR "Viet Nam") OR AD=(China OR Taiwan OR "Hong kong" OR Hongkong OR Macau OR Macao OR Beijing OR Shanghai OR Tianjin OR Chongqing OR "Inner Mongolia" OR Tibet OR Guangxi OR Sinkiang OR Ningxia OR Xinjiang OR Hebei OR Shanxi OR Liaoning OR Jilin OR Heilongjiang OR Jiangsu OR Zhejiang OR Anhui OR Fujian OR Jiangxi OR Shandong OR Henan OR Hubei OR Hunan OR Guangdong OR Hainan OR Sichuan OR Guizhou OR Yunnan OR Shaanxi OR Gansu OR Qinghai OR Japan OR Korea OR Mongolia OR Mongolian OR Afghanistan OR Afghan OR UAE OR "United Arab Emirates" OR Arab OR Arabic OR Oman OR omanis OR omani OR Azerbaijan OR Azerbaijani OR Pakistan OR Pakistani OR Palestine OR Palestinian OR Bahrain OR bahrainese OR bahraini OR Bhutan OR bhutanese OR "East Timor" OR "Timor-Leste" OR Timorese OR Philippines OR Philippine OR philippinese OR Georgia OR Georgian OR Kazakhstan OR Kyrgyzstan OR Cambodia OR Cambodian OR Qatar OR Qatari OR Kuwait OR kuwaitis OR Laos OR Lao OR Lebanon OR Lebanese OR Maldives OR maldivian* OR Malaysia OR Malaysian OR Bangladesh OR Bangladeshi OR Burma OR Myanmar OR Burmese OR Nepal OR nepalese OR Saudi Arabia OR Saudi Arabian OR Sri Lanka OR Sri Lankan OR Tajikistan OR Tajik OR Thailand OR Thai OR Turkey OR Turkish OR Turkmenistan OR Brunei OR Bruneian* OR Uzbekistan OR Singapore OR Singaporean OR Syria OR syrian* OR Armenia OR Armenian* OR Yemen OR Yemenese OR Iraq OR Iraqi OR Iran OR Iranian* OR Israel OR Israeli OR India OR Indian OR Indonesia OR indonesian* OR Jordan OR Jordanese OR Vietnam OR Vietnamese OR "Viet Nam")
#5. #3 AND #4
#6. TI=(veterinary OR rabbit* OR animal* OR mouse OR mice OR rodent* OR rat OR rats OR pig OR pigs OR porcine OR horse OR horses OR equine OR cow OR cows OR bovine OR goat OR goats OR sheep OR ovine OR canine OR dog OR dogs OR feline OR cat OR cats OR review OR "case report" OR "a case" OR 'Meta Analysis*' OR Metaanalys*)
#7. #5 NOT #6

**the Cochrane Library**

#1	MeSH descriptor: [Inflammatory Bowel Diseases] explode all trees

#2	("Inflammatory Bowel Diseas*" OR IBD OR (("Crohn-s" OR Crohn OR Crohns OR Regional* OR Granulomatous) NEAR/3 (Enteriti* OR Diseas*)) OR Ileocoliti* OR ((Granulomatous OR Terminal OR Regional OR Idiopathic OR Ulcerati* OR ulcerosa OR mucosal OR ulcerous OR Gravis*) NEAR/3 (Coliti* OR Ileiti* OR Proctocolit*))):ti,ab,kw

#3	#1 or #2

#4	(vedolizumab OR Entyvio OR "mln 02" OR kynteles OR "mln 0002" OR MLN002 OR "MLN-002" OR MLN02 OR "MLN-02" OR "LDP-02" OR "anti-alpha4beta7" OR "alpha4beta7 inhibit*" OR "alpha4beta7 antibod*" OR "Anti-α4β7" OR "α4β7 inhibit*" OR "α4β7 antibod*"):ti,ab,kw

#5	#3 and #4

#6	MeSH descriptor: [Asia] explode all trees

#7	Asia OR Asian OR Asians OR Asiatic OR China OR Chinese OR Taiwan OR Taiwanese OR "Hong kong" OR Hongkong OR Macau OR Macao OR Beijing OR Shanghai OR Tianjin OR Chongqing OR "Inner Mongolia" OR Tibet OR Guangxi OR Sinkiang OR Ningxia OR Xinjiang OR Hebei OR Shanxi OR Liaoning OR Jilin OR Heilongjiang OR Jiangsu OR Zhejiang OR Anhui OR Fujian OR Jiangxi OR Shandong OR Henan OR Hubei OR Hunan OR Guangdong OR Hainan OR Sichuan OR Guizhou OR Yunnan OR Shaanxi OR Gansu OR Qinghai OR Japan OR Japanese OR Korea OR Korean OR Mongolia OR Mongolian OR Afghanistan OR Afghan OR UAE OR "United Arab Emirates" OR Arab OR Arabic OR Oman OR omanis OR omani OR Azerbaijan OR Azerbaijani OR Pakistan OR Pakistani OR Palestine OR Palestinian OR Bahrain OR bahrainese OR bahraini OR Bhutan OR bhutanese OR "East Timor" OR "Timor-Leste" OR Timorese OR Philippines OR Philippine OR philippinese OR Georgia OR Georgian OR Kazakhstan OR Kyrgyzstan OR Cambodia OR Cambodian OR Qatar OR Qatari OR Kuwait OR kuwaitis OR Laos OR Lao OR Lebanon OR Lebanese OR Maldives OR maldivian* OR Malaysia OR Malaysian OR Bangladesh OR Bangladeshi OR Burma OR Myanmar OR Burmese OR Nepal OR nepalese OR Saudi Arabia OR Saudi Arabian OR Sri Lanka OR Sri Lankan OR Tajikistan OR Tajik OR Thailand OR Thai OR Turkey OR Turkish OR Turkmenistan OR Brunei OR Bruneian* OR Uzbekistan OR Singapore OR Singaporean OR Syria OR syrian* OR Armenia OR Armenian* OR Yemen OR Yemenese OR Iraq OR Iraqi OR Iran OR Iranian* OR Israel OR Israeli OR India OR Indian OR Indonesia OR indonesian* OR Jordan OR Jordanese OR Vietnam OR Vietnamese OR "Viet Nam"

#8	#6 or #7

#9	#5 and #8

**CNKI（期刊、学位、会议，中英文扩展：是，中文）**

(SU%=炎症性肠病+炎性肠病+克罗恩氏病+克罗恩病+局限性肠炎+局限性回肠炎+节段性肠炎+肉芽肿性肠炎+溃疡性结肠炎+溃疡结肠炎 OR TKA=炎症性肠病+炎性肠病+克罗恩氏病+克罗恩病+局限性肠炎+局限性回肠炎+节段性肠炎+肉芽肿性肠炎+溃疡性结肠炎+溃疡结肠炎) AND (SU%=生物制剂+生物药+单抗+抗体+抗肿瘤坏死因子-α+抗肿瘤坏死因子α+英夫利昔单抗+英夫利西单抗+阿达木单抗+维得利珠单抗+维多珠单抗+乌司努单抗+喜达诺+戈利木单抗+欣普尼+培塞利珠单抗+塞妥珠单抗+希敏佳+瑞莎珠单抗+那他珠单抗+"anti-TNFalpha"+ "anti-TNFa"+ "anti-TNF"+生物相似药+生物类似药+生物仿制药 OR TKA=生物制剂+生物药+单抗+抗体+抗肿瘤坏死因子-α+抗肿瘤坏死因子α+英夫利昔单抗+英夫利西单抗+阿达木单抗+维得利珠单抗+维多珠单抗+乌司努单抗+喜达诺+戈利木单抗+欣普尼+培塞利珠单抗+塞妥珠单抗+希敏佳+瑞莎珠单抗+那他珠单抗+"anti-TNFalpha"+ "anti-TNFa"+ "anti-TNF"+生物相似药+生物类似药+生物仿制药)

**万方（期刊、学位、会议）**

主题:(炎症性肠病 OR 炎性肠病 OR 克罗恩氏病 OR 克罗恩病 OR 局限性肠炎 OR 局限性回肠炎 OR 节段性肠炎 OR 肉芽肿性肠炎 OR 溃疡性结肠炎 OR 溃疡结肠炎) and 主题:("维得利珠" OR "维多珠单抗" OR "VDZ" OR "vedolizumab" OR "安吉优" OR Entyvio OR "抗整合素抗体" OR "抗α4β7")

**CBM**

( "炎症性肠病"[常用字段:智能] OR "炎性肠病"[常用字段:智能] OR "克罗恩氏病"[常用字段:智能] OR "克罗恩病"[常用字段:智能] OR "局限性肠炎"[常用字段:智能] OR "局限性回肠炎"[常用字段:智能] OR "节段性肠炎"[常用字段:智能] OR "肉芽肿性肠炎"[常用字段:智能] OR "溃疡性结肠炎"[常用字段:智能] OR "溃疡结肠炎"[常用字段:智能]) AND ( "维得利珠单抗"[常用字段:智能] OR "维得利珠"[常用字段:智能] OR "维多珠单抗"[常用字段:智能] OR "VDZ"[常用字段:智能] OR " vedolizumab "[常用字段:智能] OR "安吉优"[常用字段:智能] OR " Entyvio"[常用字段:智能] OR "抗整合素抗体"[常用字段:智能] OR "抗α4β7"[常用字段:智能])

**维普**

(M=炎症性肠病+炎性肠病+克罗恩氏病+克罗恩病+局限性肠炎+局限性回肠炎+节段性肠炎+肉芽肿性肠炎+溃疡性结肠炎+溃疡结肠炎 OR R=炎症性肠病+炎性肠病+克罗恩氏病+克罗恩病+局限性肠炎+局限性回肠炎+节段性肠炎+肉芽肿性肠炎+溃疡性结肠炎+溃疡结肠炎) AND (M=维得利珠单抗+维得利珠+维多珠单抗+VDZ+ vedolizumab +安吉优+ Entyvio+抗整合素抗体+抗α4β7 OR R=维得利珠单抗+维得利珠+维多珠单抗+VDZ+ vedolizumab +安吉优+ Entyvio+抗整合素抗体+抗α4β7)

**Supplementary 2 Specific definitions from original studies**

| **Crohn’s Disease** | | | |
| --- | --- | --- | --- |
| **Outcome** | **Definition** | **No. of studies** | **Study ID** |
| Clinical remission | CDAI≤150 | 10 | Jiang 2023  Kim 2021  Tang 2022  Tao 2022  Tian 2022  Wei 2024  Yang 2023  Yao 2022  Ye 2023  Zhou 2023 |
|  | HBI≤4 or CDAI <150 | 1 | Kopylov 2018 |
|  | HBI<5 | 2 | Chen 2023a  Lu 2022 |
| Clinical response | improvement in CDAI ≥70 | 3 | Jiang 2023  Wei 2024  Yao 2022 |
|  | improvement in CDAI >70 and HBI ≤3 | 1 | Anbarserry 2023 |
|  | improvement in CDAI ≥70 or CDAI＜150 | 1 | Tang 2022 |
|  | improvement in CDAI≥100 | 2 | Kim 2021  Zhou 2023 |
|  | improvement in HBI ≥3 | 3 | Chen 2023a  Kopylov 2018  Lu 2022 |
| Endoscopic remission | SES-CD ≤2 | 6 | Jiang 2023  Tao 2022  Tian 2022  Ye 2023  Zhou 2023  Yao 2022 |
|  | CDEIS ≤4 | 1 | Wei 2024 |
|  | no lesion in the mucosa under endoscopy | 1 | Lu 2022 |
| Endoscopic response | improvement SES-CD ≥50% | 2 | Jiang 2023  Zhou 2023 |
| **Ulcerative Colitis** | | | |
| **Outcome** | **Definition** | **No. of studies** | **Study ID** |
| Clinical remission | Mayo score ≤2 | 15 | Chen 2023b  Huai 2023  Kobayashi 2018  Kuo 2022  Lu 2022  Matsuoka 2023  Qiao 2023  Sun 2023  Tang 2023  Tian 2022  Wei 2024  Xiao 2023  Yao 2022  Zhou 2023  Ye 2023 |
|  | Mayo score ≤1 and a rectal bleeding score of 0 without additional treatment | 1 | Miyazaki 2023 |
|  | Mayo score ≤2 points with combined rectal bleeding and stool frequency subscores ≤1 | 1 | Kim 2021 |
|  | CAI ≤3 | 1 | Haga 2022 |
|  | CAI ≤4 | 1 | Yan 2023 |
|  | LI ≤3 | 1 | Saito 2021 |
|  | NR | 1 | Yoon 2024 |
|  | PRO2 or a partial Mayo Score <3 with no subscore >1 | 1 | Huang 2023 |
|  | SCCAI <2 or partial Mayo score≤2 | 1 | Kopylov 2018 |
|  | improvement in Mayo score ≥3 | 16 | Anbarserry 2023  Huai 2023  Kim 2021  Kuo 2022  Qiao 2023  Sun 2023  Tang 2023  Yao 2022  Lu 2022  Tian 2022  Xiao 2023  Zhou 2023  Chen 2023b  Miyazaki 2023  Matsuoka 2023  Wei 2024 |
| Clinical response | improvement in CAI ≥3 and an absolute score≤10 | 1 | Yan 2023 |
|  | improvement in SCCAI ≥3, or Mayo score≥2 | 1 | Kopylov 2018 |
|  | improvement in LI ≥3 and an absolute score <10 | 1 | Saito 2021 |
|  | NR | 1 | Yoon 2024 |
|  | improvement in PRO2 ≥50% | 1 | Huang 2023 |
| Endoscopic remission | MES ≤1 | 16 | Chen 2023b  Haga 2022  Huai 2023  Huang 2023  Kim 2021  Kuo 2022  Lu 2022  Qiao 2023  Saito 2021  Sun 2023  Tang 2023  Tian 2022  Wei 2024  Xiao 2023  Yao 2022  Ye 2023 |
|  | NR | 1 | Yoon 2024 |
|  | UCEIS=0 | 1 | Zhou 2023 |
| Endoscopic response | improvement in MES≥1 or UCEIS≥2 | 3 | Kim 2021  Yao 2022  Zhou 2023 |

CAI,	Clinical Activity Index; CDAI,	Crohn’s Disease Activity Index score; CDEIS,	Crohn’s Disease Endoscopic Index of Severity; HBI,	Harvey-Bradshaw Index; ID,	Identification; LI,	Lichtiger Index; MES,	Mayo Endoscopic Sub-score; PRO2,	patient-reported outcomes (rectal bleeding, and stool frequency); SCCAI,	Simple Clinical Colitis Activity Index; SES-CD,	Simple Endoscopic Score for Crohn's Disease; UCEIS,	Ulcerative Colitis Endoscopic Index of Severity.

**Supplementary 3 Quality assessment results**

**Risks of bias assessment (Crohn’s Disease -** **Newcastle-Ottawa Scale)**

| **Study ID** | | | **Liu 2022** | **Yang 2023** | **Ye 2023** |
| --- | --- | --- | --- | --- | --- |
| Selection | Representativeness of the exposed cohort | a) truly representative of the average condition in the community* | b1 | b1 | b1 |
|  |  | b) somewhat representative of the average condition in the community* |  |  |  |
|  |  | c) selected group of users eg nurses, volunteers |  |  |  |
|  |  | d) no description of the derivation of the cohort |  |  |  |
|  | Selection of the non exposed cohort | a) drawn from the same community as the exposed cohort* | a1 | a1 | b1 |
|  |  | b) drawn from a different source |  |  |  |
|  |  | c) no description of the derivation of the non exposed cohort |  |  |  |
|  | Ascertainment of exposure | a) secure record (eg surgical records)* | a1 | a1 | a1 |
|  |  | b) structured interview* |  |  |  |
|  |  | c) written self report |  |  |  |
|  |  | d) no description |  |  |  |
|  | Demonstration that outcome of interest was not present at start of study | a) yes* ¯ | a1 | a1 | a1 |
|  |  | b) no |  |  |  |
| Comparability | Comparability of cases and controls on the basis of the design or analysis | a) study controls for age* | a1b1 | a1b1 | a1b1 |
|  |  | b) study controls for any additional factor* |  |  |  |
| Outcome | Assessment of outcome | a) independent blind assessment* | b1 | b1 | b1 |
|  |  | b) record linkage* |  |  |  |
|  |  | c) self report |  |  |  |
|  |  | d) no description |  |  |  |
|  | Was follow-up long enough for outcomes to occur | a) yes* | a1 | a1 | a1 |
|  |  | b) no |  |  |  |
|  | Adequacy of follow up of cohorts | a) complete follow up - all subjects accounted for* | b1 | b1 | b1 |
|  |  | b) subjects lost to follow up unlikely to introduce bias - small number lost ≤ 20 % follow up, or description provided of those lost)* |  |  |  |
|  |  | c) follow up rate > 20% and no description of those lost |  |  |  |
|  |  | d) no statement |  |  |  |
|  |  |  | 9 | 9 | 9 |

**Risks of bias assessment (Crohn’s Disease-** **National Institutes of Health)**

| **Study ID** |  | **Anbarserry 2023** | **Chen 2023** | **Jiang 2023** | **Kim 2021** | **Kopylov 2018** | **Lu 2022** | **Tang 2022** | **Tao 2022** | **Tian 2022** | **Wei 2024** | **Yao 2022** | **Zhou 2023** |
| --- | --- | --- | --- | --- | --- | --- | --- | --- | --- | --- | --- | --- | --- |
| 1. Was the study question or objective clearly stated? | Yes | Yes | Yes | Yes | Yes | Yes | Yes | Yes | Yes | Yes | Yes | Yes | Yes |
|  | No |  |  |  |  |  |  |  |  |  |  |  |  |
|  | Other  (CD, NR, NA)* |  |  |  |  |  |  |  |  |  |  |  |  |
| 2. Were eligibility/selection criteria for the study population prespecified and clearly described? | Yes | Yes | Yes | Yes | Yes | Yes | Yes | Yes | Yes | Yes |  | Yes | Yes |
|  | No |  |  |  |  |  |  |  |  |  |  |  |  |
|  | Other  (CD, NR, NA)* |  |  |  |  |  |  |  |  |  | No |  |  |
| 3. Were the participants in the study representative of those who would be eligible for the test/service/intervention in the general or clinical population of interest? | Yes | Yes | Yes | Yes | Yes | Yes | Yes | Yes | Yes | Yes | Yes | Yes | Yes |
|  | No |  |  |  |  |  |  |  |  |  |  |  |  |
|  | Other  (CD, NR, NA)* |  |  |  |  |  |  |  |  |  |  |  |  |
| 4. Were all eligible participants that met the prespecified entry criteria enrolled? | Yes | Yes | Yes | Yes | Yes | Yes | Yes | Yes | Yes | Yes |  | Yes | Yes |
|  | No |  |  |  |  |  |  |  |  |  |  |  |  |
|  | Other  (CD, NR, NA)* |  |  |  |  |  |  |  |  |  | CD |  |  |
| 5. Was the sample size sufficiently large to provide confidence in the findings? | Yes |  |  |  |  |  |  |  |  |  |  |  |  |
|  | No |  |  |  |  |  |  |  |  |  |  |  |  |
|  | Other  (CD, NR, NA)* | CD | CD | CD | CD | CD | CD | CD | CD | CD | CD | CD | CD |
| 6. Was the test/service/intervention clearly described and delivered consistently across the study population? | Yes | Yes | Yes | Yes | Yes | Yes | Yes | Yes | Yes | Yes |  | Yes | Yes |
|  | No |  |  |  |  |  |  |  |  |  | No |  |  |
|  | Other  (CD, NR, NA)* |  |  |  |  |  |  |  |  |  |  |  |  |
| 7. Were the outcome measures prespecified, clearly defined, valid, reliable, and assessed consistently across all study participants? | Yes | Yes | Yes | Yes | Yes | Yes | Yes | Yes | Yes | Yes |  | Yes | Yes |
|  | No |  |  |  |  |  |  |  |  |  | No |  |  |
|  | Other  (CD, NR, NA)* |  |  |  |  |  |  |  |  |  |  |  |  |
| 8. Were the people assessing the outcomes blinded to the participants' exposures/interventions? | Yes |  |  |  |  |  |  |  |  |  |  |  |  |
|  | No |  |  |  |  |  |  |  |  |  |  |  |  |
|  | Other  (CD, NR, NA)* | CD | CD | CD | CD | CD | CD | CD | CD | CD | CD | CD | CD |
| 9. Was the loss to follow-up after baseline 20% or less? Were those lost to follow-up accounted for in the analysis? | Yes | Yes | Yes | Yes | Yes | Yes | Yes | Yes | Yes | Yes | Yes | Yes | Yes |
|  | No |  |  |  |  |  |  |  |  |  |  |  |  |
|  | Other  (CD, NR, NA)* |  |  |  |  |  |  |  |  |  |  |  |  |
| 10. Did the statistical methods examine changes in outcome measures from before to after the intervention? Were statistical tests done that provided p values for the pre-to-post changes? | Yes | Yes | Yes | Yes | Yes | Yes | Yes | Yes | Yes | Yes |  | Yes | Yes |
|  | No |  |  |  |  |  |  |  |  |  | No |  |  |
|  | Other  (CD, NR, NA)* |  |  |  |  |  |  |  |  |  |  |  |  |
| 11. Were outcome measures of interest taken multiple times before the intervention and multiple times after the intervention (i.e., did they use an interrupted time-series design)? | Yes | Yes | Yes | Yes | Yes | Yes | Yes | Yes | Yes | Yes |  | Yes |  |
|  | No |  |  |  |  |  |  |  |  |  | No |  | No |
|  | Other  (CD, NR, NA)* |  |  |  |  |  |  |  |  |  |  |  |  |
| 12. If the intervention was conducted at a group level (e.g., a whole hospital, a community, etc.) did the statistical analysis take into account the use of individual-level data to determine effects at the group level? | Yes |  |  |  |  |  |  |  |  |  |  |  |  |
|  | No |  |  |  |  |  |  |  |  |  |  |  |  |
|  | Other  (CD, NR, NA)* | NA | NA | NA | NA | NA | NA | NA | NA | NA | NA | NA | NA |

***CD, cannot determine; NA, not applicable; NR, not reported**

**General Guidance for Determining the Overall Quality Rating of Before-After Studies：**

The questions in the quality assessment tool were designed to help reviewers focus on the key concepts for evaluating the internal validity of a study. They are not intended to create a list from which to add up items to judge a study's quality.

Internal validity is the extent to which the outcome results reported in the study can truly be attributed to the intervention or exposure being evaluated, and not to biases, measurement errors, or other confounding factors that may result from flaws in the design or conduct of the study. In other words, what is the ability of the study to draw associative conclusions about the effects of the interventions or exposures on outcomes?

Critical appraisal of a study involves considering the risk of potential for selection bias, information bias, measurement bias, or confounding (the mixture of exposures that one cannot tease out from each other). Examples of confounding include co-interventions, differences at baseline in patient characteristics, and other issues throughout the questions above. High risk of bias translates to a rating of poor quality; low risk of bias translates to a rating of good quality. Again, the greater the risk of bias, the lower the quality rating of the study.

In addition, the more attention in the study design to issues that can help determine if there is a causal relationship between the exposure and outcome, the higher quality the study. These issues include exposures occurring prior to outcomes, evaluation of a dose-response gradient, accuracy of measurement of both exposure and outcome, and sufficient timeframe to see an effect.

Generally, when reviewers evaluate a study, they will not see a "fatal flaw," but instead will find some risk of bias. By focusing on the concepts underlying the questions in the quality assessment tool, reviewers should ask themselves about the potential for bias in the study they are critically appraising. For any box checked "no" reviewers should ask, "What is the potential risk of bias resulting from this flaw in study design or execution?" That is, does this factor lead to doubt about the results reported in the study or doubt about the ability of the study to accurately assess an association between the intervention or exposure and the outcome?

The best approach is to think about the questions in the assessment tool and how each one reveals something about the potential for bias in a study. Specific rules are not useful, as each study has specific nuances. In addition, being familiar with the key concepts will help reviewers be more comfortable with critical appraisal. Examples of studies rated good, fair, and poor are useful, but each study must be assessed on its own.

**Risks of bias assessment (Ulcerative Colitis-** **Newcastle-Ottawa Scale)**

| **Study ID** | | | **Liu 2022** | **Qiao 2023** | **Sun 2023** | **Xiao 2023** | **Ye 2023** |
| --- | --- | --- | --- | --- | --- | --- | --- |
| Selection | Representativeness of the exposed cohort | a) truly representative of the average condition in the community* | b1 | b1 | b1 | b1 | b1 |
|  |  | b) somewhat representative of the average condition in the community* |  |  |  |  |  |
|  |  | c) selected group of users eg nurses, volunteers |  |  |  |  |  |
|  |  | d) no description of the derivation of the cohort |  |  |  |  |  |
|  | Selection of the non exposed cohort | a) drawn from the same community as the exposed cohort* | a1 | a1 | a1 | a1 | a1 |
|  |  | b) drawn from a different source |  |  |  |  |  |
|  |  | c) no description of the derivation of the non exposed cohort |  |  |  |  |  |
|  | Ascertainment of exposure | a) secure record (eg surgical records)* | a1 | a1 | a1 | a1 | a1 |
|  |  | b) structured interview* |  |  |  |  |  |
|  |  | c) written self report |  |  |  |  |  |
|  |  | d) no description |  |  |  |  |  |
|  | Demonstration that outcome of interest was not present at start of study | a) yes* ¯ | a1 | a1 | a1 | a1 | a1 |
|  |  | b) no |  |  |  |  |  |
| Comparability | Comparability of cases and controls on the basis of the design or analysis | a) study controls for age* | a1b1 | a1b1 | a1b1 | a1b1 | 0 |
|  |  | b) study controls for any additional factor* |  |  |  |  |  |
| Outcome | Assessment of outcome | a) independent blind assessment* | b1 | b1 | b1 | b1 | b1 |
|  |  | b) record linkage* |  |  |  |  |  |
|  |  | c) self report |  |  |  |  |  |
|  |  | d) no description |  |  |  |  |  |
|  | Was follow-up long enough for outcomes to occur | a) yes* | a1 | a1 | a1 | a1 | a1 |
|  |  | b) no |  |  |  |  |  |
|  | Adequacy of follow up of cohorts | a) complete follow up - all subjects accounted for* | a1 | a1 | a1 | a1 | a1 |
|  |  | b) subjects lost to follow up unlikely to introduce bias - small number lost ≤ 20 % follow up, or description provided of those lost)* |  |  |  |  |  |
|  |  | c) follow up rate > 20% and no description of those lost |  |  |  |  |  |
|  |  | d) no statement |  |  |  |  |  |
|  |  |  | 9 | 9 | 9 | 9 | 7 |

**Risks of bias assessment (Ulcerative Colitis -** **National Institutes of Health)**

| **Study ID** |  | **Anbarserry 2023** | **Chen 2023** | **Haga 2022** | **Huai 2023** | **Huang 2023** | **Kim 2021** | **Kobayashi 2018** | **Kopylov 2018** | **Kuo 2022** | **Lu 2022** | **Matsuoka 2023** | **Miyazaki 2023** | **Saito 2021** | **Tang 2022** | **Tian 2022** | **Wei 2024** | **Yan 2023** | **Yao 2022** | **Yoon 2024** | **Zhou 2023** |
| --- | --- | --- | --- | --- | --- | --- | --- | --- | --- | --- | --- | --- | --- | --- | --- | --- | --- | --- | --- | --- | --- |
| 1. Was the study question or objective clearly stated? | Yes | Yes | Yes | Yes | Yes | Yes | Yes | Yes | Yes | Yes | Yes | Yes | Yes | Yes | Yes | Yes | Yes | Yes | Yes | Yes | Yes |
|  | No |  |  |  |  |  |  |  |  |  |  |  |  |  |  |  |  |  |  |  |  |
|  | Other  (CD, NR, NA)* |  |  |  |  |  |  |  |  |  |  |  |  |  |  |  |  |  |  |  |  |
| 2. Were eligibility/selection criteria for the study population prespecified and clearly described? | Yes | Yes | Yes | Yes | Yes | Yes | Yes | Yes | Yes | Yes | Yes | Yes | Yes | Yes | Yes | Yes |  | Yes | Yes |  | Yes |
|  | No |  |  |  |  |  |  |  |  |  |  |  |  |  |  |  |  |  |  |  |  |
|  | Other  (CD, NR, NA)* |  |  |  |  |  |  |  |  |  |  |  |  |  |  |  | No |  |  | No |  |
| 3. Were the participants in the study representative of those who would be eligible for the test/service/intervention in the general or clinical population of interest? | Yes | Yes | Yes | Yes | Yes | Yes | Yes | Yes | Yes | Yes | Yes | Yes | Yes | Yes | Yes | Yes | Yes | Yes | Yes | Yes | Yes |
|  | No |  |  |  |  |  |  |  |  |  |  |  |  |  |  |  |  |  |  |  |  |
|  | Other  (CD, NR, NA)* |  |  |  |  |  |  |  |  |  |  |  |  |  |  |  |  |  |  |  |  |
| 4. Were all eligible participants that met the prespecified entry criteria enrolled? | Yes | Yes | Yes | Yes | Yes | Yes | Yes | Yes | Yes | Yes | Yes | Yes | Yes | Yes | Yes | Yes |  | Yes | Yes |  | Yes |
|  | No |  |  |  |  |  |  |  |  |  |  |  |  |  |  |  |  |  |  |  |  |
|  | Other  (CD, NR, NA)* |  |  |  |  |  |  |  |  |  |  |  |  |  |  |  | CD |  |  | CD |  |
| 5. Was the sample size sufficiently large to provide confidence in the findings? | Yes |  |  |  |  |  |  |  |  |  |  |  |  |  |  |  |  |  |  |  |  |
|  | No |  |  |  |  |  |  |  |  |  |  |  |  |  |  |  |  |  |  |  |  |
|  | Other  (CD, NR, NA)* | CD | CD | CD | CD | CD | CD | CD | CD | CD | CD | CD | CD | CD | CD | CD | CD | CD | CD | CD | CD |
| 6. Was the test/service/intervention clearly described and delivered consistently across the study population? | Yes | Yes | Yes | Yes | Yes | Yes | Yes | Yes | Yes | Yes | Yes | Yes | Yes | Yes | Yes | Yes |  | Yes | Yes |  | Yes |
|  | No |  |  |  |  |  |  |  |  |  |  |  |  |  |  |  | No |  |  | No |  |
|  | Other  (CD, NR, NA)* |  |  |  |  |  |  |  |  |  |  |  |  |  |  |  |  |  |  |  |  |
| 7. Were the outcome measures prespecified, clearly defined, valid, reliable, and assessed consistently across all study participants? | Yes | Yes | Yes | Yes | Yes | Yes | Yes | Yes | Yes | Yes | Yes | Yes | Yes | Yes | Yes | Yes |  | Yes | Yes |  | Yes |
|  | No |  |  |  |  |  |  |  |  |  |  |  |  |  |  |  | No |  |  | No |  |
|  | Other  (CD, NR, NA)* |  |  |  |  |  |  |  |  |  |  |  |  |  |  |  |  |  |  |  |  |
| 8. Were the people assessing the outcomes blinded to the participants' exposures/interventions? | Yes |  |  |  |  |  |  |  |  |  |  |  |  |  |  |  |  |  |  |  |  |
|  | No |  |  |  |  |  |  |  |  |  |  |  |  |  |  |  |  |  |  |  |  |
|  | Other  (CD, NR, NA)* | CD | CD | CD | CD | CD | CD | CD | CD | CD | CD | CD | CD | CD | CD | CD | CD | CD | CD | CD | CD |
| 9. Was the loss to follow-up after baseline 20% or less? Were those lost to follow-up accounted for in the analysis? | Yes | Yes | Yes | Yes | Yes | Yes | Yes | Yes | Yes | Yes | Yes | Yes | Yes | Yes | Yes | Yes | Yes | Yes | Yes | Yes | Yes |
|  | No |  |  |  |  |  |  |  |  |  |  |  |  |  |  |  |  |  |  |  |  |
|  | Other  (CD, NR, NA)* |  |  |  |  |  |  |  |  |  |  |  |  |  |  |  |  |  |  |  |  |
| 10. Did the statistical methods examine changes in outcome measures from before to after the intervention? Were statistical tests done that provided p values for the pre-to-post changes? | Yes | Yes | Yes | Yes | Yes | Yes | Yes | Yes | Yes | Yes | Yes | Yes | Yes | Yes | Yes | Yes |  | Yes | Yes |  | Yes |
|  | No |  |  |  |  |  |  |  |  |  |  |  |  |  |  |  | No |  |  | No |  |
|  | Other  (CD, NR, NA)* |  |  |  |  |  |  |  |  |  |  |  |  |  |  |  |  |  |  |  |  |
| 11. Were outcome measures of interest taken multiple times before the intervention and multiple times after the intervention (i.e., did they use an interrupted time-series design)? | Yes | Yes | Yes |  | Yes | Yes | Yes | Yes | Yes | Yes | Yes | Yes | Yes | Yes | Yes | Yes |  |  | Yes | Yes |  |
|  | No |  |  | No |  |  |  |  |  |  |  |  |  |  |  |  | No | No |  |  | No |
|  | Other  (CD, NR, NA)* |  |  |  |  |  |  |  |  |  |  |  |  |  |  |  |  |  |  |  |  |
| 12. If the intervention was conducted at a group level (e.g., a whole hospital, a community, etc.) did the statistical analysis take into account the use of individual-level data to determine effects at the group level? | Yes |  |  |  |  |  |  |  |  |  |  |  |  |  |  |  |  |  |  |  |  |
|  | No |  |  |  |  |  |  |  |  |  |  |  |  |  |  |  |  |  |  |  |  |
|  | Other  (CD, NR, NA)* | NA | NA | NA | NA | NA | NA | NA | NA | NA | NA | NA | NA | NA | NA | NA | NA | NA | NA | NA | NA |

***CD, cannot determine; NA, not applicable; NR, not reported**

**General Guidance for Determining the Overall Quality Rating of Before-After Studies see table Risks of bias assessment (Crohn’s Disease- National Institutes of Health).**

**Table S1 Baseline characteristics of included studies**

| **Study ID** | **Continent** | **Country** | **Sample size** | **Male**  **N (%)** | **Age**  **Mean±SD**  **(years)** | **Severity of disease** | **Bio-expose**  **N (%)** | **Bio-naïven**  **N (%)** | **Duration of CD**  **Mean±SD**  **(years)** | **Concomitant medication for CD** | **Follow up (months)** |
| --- | --- | --- | --- | --- | --- | --- | --- | --- | --- | --- | --- |
| **Anbarserry 2023^1^** | Western Asia | Saudi Arabia | 4 | 3 (75.00%) | 25 ± 10.2 | moderate to severe | Pervious failure to Anti-TNF: 2 (50%) | 2 (50.00%) | 63 ± 26.6 | Concomitant immunomodulator: 0 (0.0%)  Concomitant steroid: 1 (25%) | 14 |
| **Chen 2023^2^** | Eastern Asia | China | 62 | 44 (70.97%) | 33.4±11.45 | NR | 31 (50.0%) | 31 (50.0%) | median: 731 days | 5-ASA: 19 (30.65%)  Steroids: 6 (9.68%)  IMM: 5 (8.06%)  Biologics: 31 (50.00%)  Adalimumab: 2 (3.23%)  Infliximab: 21 (33.87%)  Other TNF-a inhibitor: 4 (6.45%)  Interleukin 12/23 inhibitor: 4 (6.45%)  Other medications: 17 (27.42%) | 30 |
| **Jiang 2023^3^** | Eastern Asia | China | 22 | 12  (54.50%) | NR | mild, moderate and severe | NR | NR | NR | NR | 14 |
| **Liu 2022^4^** | Eastern Asia | China | 4 | 3 (75.00%) | NR | NR | Previous anti-TNF treatment:  3 (75.00%) | 1 (25.00%) | NR | NR | 54 |
| **Lu 2022^5^** | Eastern Asia | China | 13 | 9 (69.23%) | 38.3±11.5 | moderate and severe | 5 (38.46%) | 8 (61.54%) | median (IQR): 8.6 (5.1, 11.5) | Glucocorticoid: 1 (7.69%)  Immunosuppressant: 0 (0.00%) | median (IQR): 8.6 (5.9, 9.7) |
| **Kim 2021^6^** | Eastern Asia | China | 80 | 52 (65.00%) | NR | NR | Previous anti-TNF treatment:  1 agent: 58 (72.50%)  2 agents: 22 (27.50%)  3 agents: 0 (0.0%) | 0 | median (IQR): 11.1 (8.0–16.6) | Concomitant immunomodulator: 46 (57.50%)  Concomitant systemic corticosteroids: 16 (20.00%) | 14 |
| **Kopylov 2018^7^** | Western Asia | Israeli | 133 | 62 (46.60%) | NR | mild, moderate and severe | NR | 6 (4.50%) | NR | Systemic corticosteroids at treatment onset: 49 (36.8%)  Concomitant immunomodulators at treatment onset: 20 (15.2%) | 52 |
| **Tang 2022^8^** | Eastern Asia | China | 45 | 27 (60.00%) | NR | mild, moderate and severe | 31 (68.89%) | 14 (31.11%) | median (IQR): 60.0 (24.0, 120.0) months | Azathioprine: 1 (2.22%)  Thalidomide: 1 (2.22%) | 52 |
| **Tao 2022^9^** | Eastern Asia | China | 30 | 21 (70.00%) | 31.6±11.5 | NR | 0 (0.00%) | 30 (100.00%) | median (IQR): 21.50 (12.50, 30.25) months | NR | 52 |
| **Tian 2022^10^** | Eastern Asia | China | 14 | 10 (71.43%) | NR | moderate and severe | 9 (64.29%) | 5 (35.71%) | 4 (0.17,10) | NR | 14 |
| **Wei 2024^11,12^** | Eastern Asia | China | 127 | NR | 33.4 ± 14.6 | NR | 63 (49.6%) | 64 (50.40%) | disease duration: 3.1 | NR | 1 year |
| **Yang 2023^13^** | Eastern Asia | China | 150 | 95 (63.33%) | 34.6 ± 10.9 | NR | 84 (56.00%) | 66 (44.00%) | 71.7±57.3 months | NR | 52 |
| **Yao 2022^14^** | Eastern Asia | China | 39 | 26 (66.7%) | 30.3±9.8 | NR | 23 (59.0%) | NR | 80.1±57.7months | NR | 22 |
| **Ye 2023^15^** | Eastern Asia | China | 97 | 53 (54.64%) | 31.6±10.3NR | mild, moderate and severe | NR | NR | NR | NR | 54 |
| **Zhou 2023^16^** | Eastern Asia | China | 7 | 3 (42.86%) | NR | mild, moderate to severe | 4 (57.14%) | NR | NR | NR | 14 |
| **Study ID** | **Continent** | **Country** | **Sample size** | **Male**  **N (%)** | **Age**  **Mean±SD**  **(years)** | **Severity of disease** | **Bio-expose**  **N (%)** | **Bio-naïven**  **N (%)** | **Duration of UC**  **Mean±SD**  **(years)** | **Concomitant medication for UC** | **Follow up (months)** |
|  |  |  |  |  |  |  |  |  |  |  |  |
| **Anbarserry 2023^1^** | Western Asia | Saudi Arabia | 12 | 4 (33.33%) | 31.8 ± 11.6 | moderate to severe | 0 (0.0%) | 12 (100.00%) | 39.8±54.3 | Concomitant immunomodulator: 3 (25.00%)  Concomitant steroid: 4 (33.30%) | 14 |
| **Chen 2023^2^** | Eastern Asia | China | 245 | 149 (60.82%) | 45.6±14.79 | NR | 26 (10.61%) | NR | median: 1035 days | NR | 72 |
| **Haga 2022^17^** | Eastern Asia | Japan | 87 | 47 (54.02%) | 45.2 ± 14.0 | moderate to severe | Prior bio use  1: 22 (25.3%)  2: 11 (12.6%)  3: 1 (1.2%) | NR | 9.4±7.5 | 5-Aminosalicylate: 78 (89.70%)  Prednisolone: 36 (41.40%)  Azathioprine: 33 (37.90%) | 52 |
| **Huai 2023^18^** | Eastern Asia | China | 25 | 11 (44.00%) | NR | moderate and severe | 5 (20.00%) | 20 (80.00%) | 6-486 months | NR | 22 |
| **Huang 2023^19^** | Eastern Asia | China | 64 | 36 (56.30%) | 45.1 ± 13.3 | moderate to severe | Anti-TNF agents: 8 (12.50%) | NR | mmedian (IQR): 5.3 (2.0, 8.9) | Concomitant medications  Corticosteroids: 8 (12.50%)  Tofacitinib: 2 (3.10%) | 52 |
| **Kim 2021^6^** | Eastern Asia | Korea | 78 | 44 (56.40%) | NR | NR | Previous anti-TNF treatment  1 agent: 49 (62.80%)  2 agents: 25 (32.10%)  3 agents: 4 (5.10%) | 0 | median (IQR): 5.3 (2.4–7.8) | Concomitant immunomodulator: 33 (42.30%)  Concomitant systemic corticosteroids: 31 (39.70%) | 14 |
| **Kobayashi 2018^20^** | Eastern Asia | Japan | 6 | 5  (83.30%) | Mean: 44.3 | NR | NR | NR | NR | NR | 44-238 days |
| **Kopylov 2018^7^** | Western Asia | Israeli | 60 | 30 (50.00%) | NR | mild, moderate and severe | NR | 9 (15.10%) | NR | Systemic corticosteroids at treatment onset: 9 (15.10%)  Concomitant immunomodulators at treatment onset: 14 (23.30%) | 52 |
| **Kuo 2022^21^** | Eastern Asia | China | 37 | 19 (51.40%) | 38.6 ± 16.8 | moderate to severe | Previous exposure to anti-TNF therapy 7 (18.90%)  Adalimumab: 5 (13.50%) | NR | 0.70 ± 1.70 | NR | 52 |
| **Liu 2022^4^** | Eastern Asia | China | 11 | 3  (27.27%) | 45.8±12.3 | mild, moderate and severe | Previous anti-TNF treatment:  1 (9.09%) | 10 (80.91%) | NR | NR | 54 |
| **Lu 2022^5^** | Eastern Asia | China | 65 | 35  (53.80%) | 41.0±12.1 | moderate and severe | 21 (32.30%) | NR | median (IQR): 7.3 (3.8, 12.6) | Glucocorticoid: 21 (32.30%)  Immunosuppressant: 5 (7.70%) | median (IQR): 8.8 (6.9, 10.5) months |
| **Matsuoka 2023^22^** | Eastern Asia | Japan | 268 | 160 (59.70%) | 43.60 ± 17.55 | moderate to severe | 116 (43.3%) | 152 (56.7%) | 9.10 ± 7.76 | NR | 54 |
| **Miyazaki 2023^23^** | Eastern Asia | Japan | 42 | 16 (38.10%) | NR | moderate to severe | Prior use of biologics: 19 (45.2) | NR | median (range): 6 (0–37) | Systemic corticosteroids: 16 (38.10)  5-aminosalicylates: 11 (26.20)  Immunosuppressants: 12 (28.60) | 22 |
| **Qiao 2023^24^** | Eastern Asia | China | 36 | 23  (63.89%) | 40.11±9.00 | moderate and severe | NR | NR | 2.03±0.57 | NR | 30 |
| **Saito 2021^25^** | Eastern Asia | Japan | 52 | 36 (69.20%) | NR | NR | Prior anti-TNF use:  1: 10 (19.20%)  2: 4 (7.70%)  3: 1 (1.90%) | NR | median (IQR): 5 (1.6, 8.3) | 5-aminosalicylic acid: 33 (61.10%)  Azathioprine: 14 (26.90%)  Prednisolone: 13 (25.00%) | 24 |
| **Sun 2023^26^** | Eastern Asia | China | 30 | 13 (43.33%) | 44.47±14.71 | moderate and severe | NR | NR | median duration of disease 6.5years | NR | 14 |
| **Tang 2023^27^** | Eastern Asia | China | 81 | 50  (61.73 %) | NR | mild, moderate and severe | 58 (71.60%) | 23 (28.40%) | median (IQR): 42.5 (22.5, 94.7) months | Glucocorticoid: 17 (21.00%)  5-aminosalicylic acid: 41 (50.60%)  Thalidomide: 1 (1.23%) | 52 |
| **Tian 2022^10^** | Eastern Asia | China | 24 | 11  (45.83%) | NR | moderate and severe | 5 (20.83%) | 19 (79.17%) | 4.5 (0.08,15) | NR | 14 |
| **Wei 2024^11,12^** | Eastern Asia | China | 147 | NR | 42.40 ± 14.30 | NR | 43 (29.30%) | 104 (70.70%) | median disease duration (years): 3.9 | NR | 1 year |
| **Xiao 2023^28^** | Eastern Asia | China | 37 | 19 (51.35%) | 36.8±11.8 | moderate and severe | 9 (24.32%) | 28 (75.68%) | 2.5 (1.8, 5.0) | Glucocorticoid: 3 (8.11%)  5‑aminosalicylic acid: 21 (56.76%) | 72 |
| **Yao 2022^13^** | Eastern Asia | China | 13 | 6 (46.20%) | 40.60±18.10 | mild, moderate and severe | 4 (30.80%) | NR | 101.80±105.20 months | NR | 22 |
| **Ye 2023^14^** | Eastern Asia | China | 40 | 22  (55.00%) | 37.50±14.70 | mild, moderate and severe | NR | NR | NR | NR | 54 |
| **Yoon 2022^29^** | Eastern Asia | Korea | 27 | NR | NR | NR | NR | NR | NR | NR | 54 |
| **Yan 2023^30^** | Eastern Asia | China | 74 | 50 (67.60%) | NR | NR | Anti-TNF: 10 (13.50%) | NR | median (IQR): 4.5 (2.0–9.3) | 5-ASA: 67 (90.50%)  Corticosteroids: 24 (32.40%) | 14 |
| **Zhou 2023^15^** | Eastern Asia | China | 7 | 2  (28.57%) | NR | mild, moderate to severe | 1 (14.29%) | NR | NR | NR | 14 |

*5-ASA, 5-Amino salicylic acid; CD, Crohn’s disease; IMM, Immune Mediated Mechanisms; IQR, Interquartile Range; NR, not reported; SD, Standard Deviation; TNF, tumor necrosis factor; UC, Ulcerative Colitis.*

**Reference**

1.	Anbarserry D, Mosli M, Qari Y, Saadah O, Bokhary R, Esmat A, et al. The use of therapeutic drug monitoring for early identification of vedolizumab response in Saudi Arabian patients with inflammatory bowel disease. Scientific reports. 2023;13(1):1771.

2.	Chen BL, Xie L, Liu YM, Chen MH. Clinical Effectiveness of Vedolizumab in Chinese Crohn's Disease Patients: Results From the VALUE Study. American Journal of Gastroenterology. 2023;118(10):S211-S3.

3.	Jiang LJ, Chen L, Chen YF, Chen YR, Chen H. Observation on the efficacy of vedolizumab in the treatment of patients with active Crohn′s disease. Chinese Journal of Digestion. 2023;43(6):395-400.

4.	Liu SX, Zhong W, Xia ZS, Zhong YQ. Clinical efficacy of vedolizumab for inflammatory bowel disease. New Medicine. 2022;53(10):767-73.

5.	Lu B, Liu ZS, Zheng WY, Bai XY, Yang H, Qian JM. Short-term efficacy and safety of vedolizumab in patients with inflammatory bowel disease. National Medical Journal of China. 2022;102(42):3388-94.

6.	Kim J, Yoon H, Kim N, Lee KM, Jung SA, Choi CH, et al. Clinical Outcomes and Response Predictors of Vedolizumab Induction Treatment for Korean Patients With Inflammatory Bowel Diseases Who Failed Anti-TNF Therapy: A KASID Prospective Multicenter Cohort Study. Inflammatory Bowel Diseases. 2021;27(12):1931-41.

7.	Kopylov U, Avni-Biron I, Ron Y, Koslowsky B, Waterman M, Daher S, et al. Effectiveness and safety of vedolizumab for maintenance treatment in inflammatory bowel disease-The Israeli real world experience. Digestive and liver disease : official journal of the Italian Society of Gastroenterology and the Italian Association for the Study of the Liver. 2019;51(1):68-74.

8.	Tang J, Deng J, Huang ZC, Huang ZP, Guo HL, Diao N, et al. The real-world efficacy and safety of vedolizumab in the treatment of active Crohn′s disease: a single center retrospective study. Chinese Journal of Inflammatory Bowel Diseases. 2022;06(3):217-22.

9.	Tao QH, Lv H. The predictive value of fecal calprotectin level for the efficacy of vidalizumab in the treatment of patients with Crohn's disease. Modern Practical Medicine. 2022;34(12):1591-4+682.

10.	Tian XY. Efficacy analysis of vedolizumab in the treatment of moderate to severe ulcerative colitis and Crohn's disease [Master]2022.

11.	Wei SC, Lin WC, Chang CH, Tu CH, Feng IC, Shieh MJ, et al. Real-world evidence on effectiveness and safety of vedolizumab therapy for inflammatory bowel disease in Taiwan: Results from the TSIBD registry (VIOLET Study). Journal of Crohns & Colitis. 2021;15:S428-S30.

12.	Lin WC, Tai WC, Chang CH, Tu CH, Feng IC, Shieh MJ, et al. Real-World Evidence of Effectiveness and Safety of Vedolizumab for Inflammatory Bowel Disease in Taiwan: A Prospective Nationwide Registry (VIOLET) Study. Inflamm Bowel Dis. 2023;29(11):1730-40.

13.	Yang H, Huang Z, Li M, Zhang H, Fu L, Wang X, et al. Comparative effectiveness of ustekinumab vs. vedolizumab for anti-TNF-naïve or anti-TNF-exposed Crohn's disease: a multicenter cohort study. EClinicalMedicine. 2023;66:102337.

14.	Yao JY, Peng X, Liu T, Zhao JZ, Zhang M, Hu PJ, et al. Short-term efficacy and safety of vedolizumab in the treatment of inflammatory bowel disease: A single-center real-world study. Journal of Clinical and Pathological Research. 2022;42(08):1841-6.

15.	Ye ML, Hu T, Xu LJ, Zhang LP, Yin J, Yu Q, et al. The diagnostic and evaluation value of plasma interleukin 9 in the mucosal healing in patients with inflammatory bowel disease treated with biological agents. National Medical Journal of China. 2023;103(19):1483-9.

16.	Zhou SM, Tian Y, Guo XJ, Huang Q, Song ZJ, Jiang X, et al. Efficacy and safety of Vedolizumab in the treatment of inflammatory bowel disease:a single-center real-world study. Chinese Journal of Gastroenterology and Hepatology. 2023;32(09):977-81.

17.	Haga K, Shibuya T, Osada T, Sato S, Fukuo Y, Kobayashi O, et al. Early Clinical Remission Is a Predictor of Long-Term Remission with the Use of Vedolizumab for Ulcerative Colitis. Biomedicines. 2022;10(10).

18.	Huai MX, Duan XY, Ge WS. Efficacy of vedolizumab in patients with moderate to severe ulcerative colitis. Journal of Zhengzhou University(Medical Sciences). 2023;58(01):117-20.

19.	Huang K, Liu J, Xia W, Tian C, Yao L, Cao Q, et al. Effectiveness and safety of vedolizumab for ulcerative colitis: a single-center retrospective real-world study in China. Frontiers in Pharmacology. 2023;14:1188751.

20.	Kobayashi K, Suzuki Y, Watanabe K, Oda K, Mukae M, Yamada A, et al. A Phase 1, Multiple-Dose Study of Vedolizumab in Japanese Patients With Ulcerative Colitis. Journal of clinical pharmacology. 2019;59(2):271-9.

21.	Kuo CJ, Le PH, Tai WC, Wu KL, Yen HH, Yen CW, et al. The effectiveness and safety of vedolizumab induction for moderate to severe ulcerative colitis for Asia patient: A real practice observational study. Journal of the Formosan Medical Association = Taiwan yi zhi. 2022;121(9):1689-95.

22.	Matsuoka K, Hisamatsu T, Mikami Y, Yamamoto T, Motoya S, Shinzaki S, et al. Safety and Effectiveness of Vedolizumab in Patients with Moderate-to-Severe Ulcerative Colitis: An Interim Analysis of a Japanese Post-Marketing Surveillance Study. Advances in Therapy. 2023;40(6):2902-14.

23.	Miyazaki H, Hoshi N, Ishida T, Nishioka C, Ouchi S, Shirasaka D, et al. Association of CD4-positive cell infiltration with response to vedolizumab in patients with ulcerative colitis. Scientific reports. 2023;13(1):20262.

24.	Qiao HW. Efficacy of verdelezumab combin with glucocorticoid in the treatment of moderate and severe ulcerative colitis [master's thesis]2023.

25.	Saito D, Matsuura M, Ozaki R, Tokunaga S, Minowa S, Mitsui T, et al. Clinical response of vedolizumab at week 6 predicted endoscopic remission at week 24 in ulcerative colitis. JGH open : an open access journal of gastroenterology and hepatology. 2021;5(9):1056-62.

26.	Sun CX. Vedolizumab and infliximab in the treatment of ulcerative colitis: clinical efficacy analysis [master's thesis]2023.

27.	Tang J, Huang ZP, Deng J, Huang ZC, Diao N, Yang HS, et al. The efficacy and safety of vedolizumab in the treatment of 81 cases of active ulcerative colitis: a single center retrospective study. Chinese Journal of Digestion. 2023;43(2):117-21.

28.	Xiao HY, Rao SY, Zhang DL, Ma GL, Xu Y, Jiang Y. Explore the influence of vitamin D supplementation on clinical efficacy and drug retention rate of vedolizumab in patients with ulcerative colitis. National Medical Journal of China. 2023;103(23):1759-66.

29.	Yoon A, Kim K, Oh K, Kim ES, Kim EH, Kim S, et al. Compositional differences of gut microbiome using taxonomic profiling in patients with ulcerative colitis after treatment with vedolizumab. Journal of Crohns & Colitis. 2022;16:I615-I6.

30.	Yan J, Liu A, Fang L, Wu J, Ding X, Xu Y. The ulcerative colitis endoscopic index of severity score is superior to reflecting long-term prognosis in ulcerative colitis patients treated with vedolizumab. Medicine. 2023;102(44):e35799.

**Table S2 Results for Asian population**

| **Outcomes** | **Number of studies** | | **Number of patients** | | **Outcome data proportion [95% confidence interval]** |
| --- | --- | --- | --- | --- | --- |
| **Crohn’s disease** | | | | | |
| **Induction phase** | | | | | |
| CRP normalization rate | | 1 | | 13 | 61.54% [31.58%, 86.14%] |
| Treatment persistent rate | | 1 | | 102 | 95.10% [88.93%, 98.39%] |
| **Medium term** | | | | | |
| CRP normalization rate | | - | | - | - |
| Treatment persistent rate | | 1 | | 76 | 94.00% [88.66%, 99.34%] |
| **Long term** | | | | | |
| CRP normalization rate | | - | | - | - |
| Treatment persistent rate | | 1 | | 34 | 81.80% [68.83%, 94.77%] |
| **Ulcerative Colitis** | | | | | |
| **Induction phase** | | | | | |
| Faecal calprotectin normalization | | 1 | | 22 | 36.36% [17.2%, 59.34%] |
| CRP normalization rate | | 2 | | 79 | 64.47% [43.59%, 85.34%] |
| Treatment persistent rate | | 1 | | 128 | 96.88% [92.19%, 99.14%] |
| **Medium term** | | | | | |
| Faecal calprotectin normalization | | - | | - | - |
| CRP normalization rate | | - | | - | - |
| Treatment persistent rate | | 1 | | 98 | 94.40% [89.85%, 98.95%] |
| **Long term** | | | | | |
| Faecal calprotectin normalization | | 1 | | 15 | 33.33% [11.82%, 61.62%] |
| CRP normalization rate | | 1 | | 11 | 63.64% [30.79%, 89.07%] |
| Treatment persistent rate | | 1 | | 31 | 93.55% [78.58%, 99.21%] |
| *Induction term: 6-14 weeks; medium term: 22-30 weeks; long term: over than 48 or 52 weeks;*  *CRP stands for C-reaction protein.* | | | | | |

**Table S3 Effectiveness in patients treated with vedolizumab in various follow-up periods-sensitivity analysis**

| **Outcomes** | **Crohn’s Disease** | | | | **Ulcerative Colitis** | | | |
| --- | --- | --- | --- | --- | --- | --- | --- | --- |
|  | **Number of studies** | **Number of patients** | **Outcome data proportion [95% confidence interval]** | **Heterogeneity [*I^2^]*** | **Number of studies** | **Number of patients** | **Outcome data proportion [95% confidence interval]** | **Heterogeneity [*I^2^]*** |
| **Induction phase** | | | | | | | | |
| Clinical remission rate | 4 | 100 | 70.04% [45.48%, 94.61%] | 86% | 9 | 436 | 39.56% [23.27%, 55.84%] | 92% |
| Clinical response rate  Decrease in CDAl≥100  Decrease in CDAl≥70 | 2  1 | 66  22 | 67.13% [34.86%, 99.40%]  95.45% [77.16%, 99.88%] | 80%  — | 6 | 271 | 67.48% [55.68%, 79.29%] | 77% |
| Endoscopic remission rate | 3 | 41 | 6.66% [0.00%, 16.76%] | 27% | 6 | 249 | 27.98% [18.55%, 37.40%] | 64% |
| **Medium term** | | | | | | | | |
| Clinical remission rate | 4 | 318 | 53.72% [43.75%, 63.68%] | 67% | 6 | 184 | 43.98% [22.54%, 65.42%] | 90% |
| Clinical response rate  Decrease in CDAl≥70 | 2 | 123 | 71.58% [40.97%, 100.00%] | 94% | 3 | 74 | 59.95% [17.28%, 100.00%] | 95% |
| Endoscopic remission rate | 1 | 17 | 29.41% [10.31%, 55.96%] | — | 3 | 121 | 47.87% [34.51%, 61.24%] | 51% |
| **Long term** | | | | | | | | |
| Clinical remission rate | 5 | 296 | 51.28% [35.27%, 67.30%] | 86% | 4 | 420 | 60.64% [55.57%, 66.17%] | 11% |
| Clinical response rate  Decrease in CDAl≥70 | 1 | 56 | 57.14% [43.22%, 70.29%] | — | 1 | 43 | 69.77% [53.87%, 82.82%] | — |
| Endoscopic remission rate | 2 | 57 | 40.27% [27.56%, 52.98%] | 0% | 3 | 34 | 50.61% [25.2%, 76.02%] | 51% |
| *Induction term: 6-14 weeks; medium term: 22-30 weeks; long term: over than 48 or 52 weeks.*  *These include·a·CDAI≤150 for clinical remission·in CD;·a Mayo·score·≤2 with no subscore >1 for clinical remission in UC; a decrease in CDAI≥100 or≥70 for clinical response in CD; a decrease in Mayo-score≥3 and ≥30% from baseline with a rectal bleeding subscore decrease of ≥1 or absolute score ≤1 in·UC; a SES-CD ≤2 for endoscopic remission in CD. Mayo endoscopic score of 0 for endoscopic remission for UC.* | | | | | | | | |

**Table S4 Summary of safety outcomes of Crohn’s Disease in Asian patients receiving VDZ**

| **AEs** | **Study ID** | **Events** | **Total** | **Percentage** | **Time point** |
| --- | --- | --- | --- | --- | --- |
| **AE** | | | | **Range: 6.67%-61.54%** | |
|  | Yang 2023 | 10 | 150 | 6.67% | 52 weeks |
|  | Lu 2022 | 8 | 13 | 61.54% | 14 weeks |
| **Mild AE** | | | | **Range: 79.03%** | |
|  | Chen 2023 | 49 | 62 | 79.03% | 30 weeks |
| **SAE** | | | | **Range: 0.00%** | |
|  | Liu 2022 | 0 | 4 | 0.00% | 54 weeks |
| **Discontinuation** | | | | | |
| Any AEs leading to discontinuation | | | | **Range: 6.45%** | |
|  | Chen 2023 | 4 | 62 | 6.45% | 30 weeks |
| Specific reasons for discontinuation | | | | | |
| Lack of response | | | | **Range: 1.25%** | |
|  | Kim 2021 | 1 | 80 | 1.25% | 6 weeks |
|  | Kim 2021 | 1 | 80 | 1.25% | 14 weeks |
| Perianal fistula–associated mucinous adenocarcinoma | | | | **Range: 1.25%** | |
|  | Kim 2021 | 1 | 80 | 1.25% | 6 weeks |
| Joint pain | | | | **Range: 1.25%-2.22%** | |
|  | Kim 2021 | 1 | 80 | 1.25% | 6 weeks |
|  | Tang 2022 | 1 | 45 | 2.22% | 52 weeks |
| **Serious infection** | | | | | |
| Serious infection | | | | **Range: 0.00%** | |
|  | Tian 2022 | 0 | 14 | 0.00% | 14 weeks |
| Line sepsis | | | | **Range: 0.75%** | |
|  | Kopylov 2018 | 1 | 133 | 0.75% | 52 weeks |
| Sepsis (resulting in death) | | | | **Range: 0.00%** | |
|  | Kopylov 2018 | 0 | 133 | 0.00% | 52 weeks |
| **CDI** | | | | | |
|  |  |  |  | **Range: 2.00%** | |
|  | Yang 2023 | 3 | 150 | 2.00% | 52 weeks |
| **Hemopathy** | | | | | |
| Idiopathic thrombocytopenic purpura | | | | **Range: 0.75%** | |
|  | Kopylov 2018 | 1 | 133 | 0.75% | 52 weeks |
| **Opportunistic infection** | | | | | |
| New active tuberculosis and hepatitis B virus infection | | | | **Range: 0.00%** | |
|  | Tang 2022 | 0 | 45 | 0.00% | 52 weeks |
| **Infusion reaction** | | | | **Range: 0.00%** | |
|  | Kopylov 2018 | 0 | 133 | 0.00% | 14 weeks |
|  | Tian 2022 | 0 | 12 | 0.00% | 52 weeks |
| *AE, adverse event; CDI, clostridium difficile infection; SAE, serious adverse events* | | | | | |

**Table S5 Summary of safety outcomes of Ulcerative Colitis in Asian patients receiving VDZ**

| **AEs** | **Study ID** | **Events** | **Total** | **Percentage** | **Time point** |
| --- | --- | --- | --- | --- | --- |
| **AE** | | | | **Range: 29.69%-75.38%** | |
|  | Huang 2023 | 19 | 64 | 29.69% | 52 weeks |
|  | Lu 2022 | 49 | 65 | 75.38% | 14 weeks |
| **Mild AE** |  |  |  | **Range: 70.14%** | |
|  | Chen 2023 | 148 | 211 | 70.14% | 72 weeks |
| **SAE** | | | | **Range: 0.00%-1.56%** | |
|  | Liu 2022 | 0 | 11 | 0.00% | 54 weeks |
|  | Huai 2023 | 0 | 25 | 0.00% | 22 weeks |
|  | Huang 2023 | 1 | 64 | 1.56% | 52 weeks |
| **Discontinuation** | | | | | |
| Any AEs leading to discontinuation | | | | **Range: 0.00%-1.90%** | |
|  | Kuo 2022 | 0 | 37 | 0.00% | 52 weeks |
|  | Yan 2023 | 1 | 74 | 1.35% | 14 weeks |
|  | Lu 2022 | 1 | 65 | 1.54% | 14 weeks |
|  | Chen 2023 | 4 | 211 | 1.90% | 72 weeks |
| Specific reasons for discontinuation | | | | | |
| Lack of response | | | | **Range: 1.28%-5.13%** | |
|  | Kim 2021 | 1 | 78 | 1.28% | 6 weeks |
|  | Kim 2021 | 4 | 78 | 5.13% | 14 weeks |
| Enlarged pulmonary nodule | | | | **Range: 1.60%** | |
|  | Huang 2023 | 1 | 64 | 1.60% | 52 weeks |
| Recurrence of symptoms of intestinal Aeromonas infection | | | | **Range: 1.23%** | |
|  | Tang 2023 | 1 | 81 | 1.23% | 52 weeks |
| **TRAE** | | | | **Range: 0.00%-13.89%** | |
|  | Kobayashi 2018 | 0 | 6 | 0.00% | 34 weeks |
|  | Matsuoka 2023 | 11 | 268 | 4.10% | 6 weeks |
|  | Qiao 2023 | 5 | 36 | 13.89% | 30 weeks |
| **Mild TRAE** | | | | **Range: 16.22%** | |
|  | Kuo 2022 | 6 | 37 | 16.22% | 52 weeks |
| **STRAE** | | | | **Range: 0.00%** | |
|  | Kuo 2022 | 0 | 37 | 0.00% | 52 weeks |
| **TEAE** | | | | **Range: 100.00%** | |
|  | Kobayashi 2018 | 6 | 6 | 100.00% | 34 weeks |
| **STEAE** | | | | **Range: 16.67%** | |
|  | Kobayashi 2018 | 1 | 6 | 16.67% | 34 weeks |
| **Drug-related STEAEs** | | | | **Range: 16.67%** | |
|  | Kobayashi 2018 | 1 | 6 | 16.67% | 34 weeks |
| **TEAEs leading to discontinuation** | | | | **Range: 0.00%** | |
|  | Kobayashi 2018 | 0 | 6 | 0.00% | 34 weeks |
| **Drug-related STEAEs leading to discontinuation** | | | | **Range: 0.00%** | |
|  | Kobayashi 2018 | 0 | 6 | 0.00% | 34 weeks |
| **Serious infection** | | | | | |
| Serious infection | | | | **Range: 0.00%** | |
|  | Tian 2022 | 0 | 24 | 0.00% | 14 weeks |
| Pneumonia | | | | **Range: 0.37%-1.15%** | |
|  | Haga 2022 | 1 | 87 | 1.15% | 52 weeks |
|  | Matsuoka 2023 | 1 | 268 | 0.37% | 6 weeks |
| Line sepsis | | | | **Range: 0.00%** | |
|  | Kopylov 2018 | 0 | 60 | 0.00% | 52 weeks |
| Listeria monocytogenes sepsis | | | | **Range: 2.70%** | |
|  | Kuo 2022 | 1 | 37 | 2.70% | 52 weeks |
| Sepsis (resulting in death) | | | | **Range: 1.67%** | |
|  | Kopylov 2018 | 1 | 60 | 1.67% | 52 weeks |
| **CDI** | | | | **Range: 12.35%** | |
|  | Tang 2023 | 10 | 81 | 12.35% | 52 weeks |
| **Hemopathy** | | | | | |
| Idiopathic thrombocytopenic purpura | | | | **Range: 0.00%** | |
|  | Kopylov 2018 | 0 | 60 | 0.00% | 52 weeks |
| **Opportunistic infection** | | | | | |
| Tuberculosis | | |  | **Range: 0.00%** | |
|  | Huang 2023 | 0 | 64 | 0.00% | 52 weeks |
|  | Kuo 2022 | 0 | 37 | 0.00% | 52 weeks |
|  | Tang 2023 | 0 | 81 | 0.00% | 52 weeks |
|  | Tian 2022 | 0 | 24 | 0.00% | 14 weeks |
| Hepatitis B virus infection | | | | **Range: 0.00%** | |
|  | Huang 2023 | 0 | 64 | 0.00% | 52 weeks |
|  | Kuo 2022 | 0 | 37 | 0.00% | 52 weeks |
|  | Tang 2023 | 0 | 81 | 0.00% | 52 weeks |
| Aeromonas intestinalis infection | | | | **Range: 1.23%** | |
|  | Tang 2023 | 1 | 81 | 1.23% | 52 weeks |
| Cytomegalovirus infection | | | | **Range: 0.00%-3.33%** | |
|  | Sun 2023 | 1 | 30 | 3.33% | 14 weeks |
|  | Tian 2022 | 0 | 24 | 0.00% | 14 weeks |
| Herpes zoster | | | | **Range: 0.37%-2.78%** | |
|  | Matsuoka 2023 | 1 | 268 | 0.37% | 6 weeks |
|  | Qiao 2023 | 1 | 36 | 2.78% | 30 weeks |
| **Pyoderma gangrenosum** | | | | **Range: 1.15%** | |
|  | Haga 2022 | 0 | 87 | 1.15% | 52 weeks |
| **Infusion reaction** | | | | **Range: 0.00%-1.70%** | |
|  | Huang 2023 | 0 | 64 | 0.00% | 52 weeks |
|  | Kopylov 2018 | 1 | 60 | 1.70% | 52 weeks |
|  | Sun 2023 | 0 | 30 | 0.00% | 14 weeks |
|  | Tang 2023 | 0 | 81 | 0.00% | 52 weeks |
|  | Tian 2022 | 0 | 24 | 0.00% | 14 weeks |
| **Thrombosis** | | | | | |
| Cerebral infarction | | | | **Range: 1.15%** | |
|  | Haga 2022 | 1 | 87 | 1.15% | 52 weeks |
| Deep vein thrombosis | | | | **Range: 1.15%** | |
|  | Haga 2022 | 1 | 87 | 1.15% | 52 weeks |
| *AE, adverse event; CDI, clostridium difficile infection; SAE, serious adverse events; STEAE, serious treatment-emergent adverse events; STRAE, serious treatment-related adverse events; TEAE, treatment-emergent adverse events; TRAE, treatment-related adverse event* | | | | | |

**Table S6 Summary of safety outcomes of Crohn’s Disease in Chinese patients receiving VDZ**

| **AEs** | **Study ID** | **Events** | **Total** | **Percentage** | **Time point** |
| --- | --- | --- | --- | --- | --- |
| **AE** | | | | **Range: 6.67%-61.54%** | |
|  | Yang 2023 | 10 | 150 | 6.67% | 52 weeks |
|  | Lu 2022 | 8 | 13 | 61.54% | 14 weeks |
| **Mild AE** | | | | **Range: 79.03%** | |
|  | Chen 2023 | 49 | 62 | 79.03% | 30 weeks |
| **SAE** | | | | **Range: 0.00%** | |
|  | Liu 2022 | 0 | 4 | 0.00% | 54 weeks |
| **Discontinuation** | | | | | |
| Any AEs leading to discontinuation | | | | **Range: 6.45%** | |
|  | Chen 2023 | 4 | 62 | 6.45% | 30 weeks |
| Specific reasons for discontinuation | | | | | |
| Joint pain | | | | **Range: 2.22%** | |
|  | Tang 2022 | 1 | 45 | 2.22% | 52 weeks |
| **Serious infection** | | | | | |
| Serious infection | | | | **Range: 0.00%** | |
|  | Tian 2022 | 0 | 14 | 0.00% | 14 weeks |
| **CDI** | | | | **Range: 2.00%** | |
|  | Yang 2023 | 3 | 150 | 2.00% | 52 weeks |
| **Opportunistic infection** | | | | | |
| New active tuberculosis and hepatitis B virus infection | | | | **Range: 0.00%** | |
|  | Tang 2022 | 0 | 45 | 0.00% | 52 weeks |
| *AE, adverse event; CDI, clostridium difficile infection; SAE, serious adverse events* | | | | | |

**Table S7 Summary of safety outcomes of Ulcerative Colitis in Chinese patients receiving VDZ**

| **AEs** | **Study ID** | **Events** | **Total** | **Percentage** | **Time point** |
| --- | --- | --- | --- | --- | --- |
| **AE** | | | | **Range: 29.69%-75.38%** | |
|  | Huang 2023 | 19 | 64 | 29.69% | 52 weeks |
|  | Lu 2022 | 49 | 65 | 75.38% | 14 weeks |
| **Mild AE** | | | | **Range: 70.14%** | |
|  | Chen 2023 | 148 | 211 | 70.14% | 72 weeks |
| **SAE** | | | | **Range: 0.00%-1.56%** | |
|  | Liu 2022 | 0 | 11 | 0.00% | 54 weeks |
|  | Huai 2023 | 0 | 25 | 0.00% | 22 weeks |
|  | Huang 2023 | 1 | 64 | 1.56% | 52 weeks |
| **Discontinuation** | | | | | |
| Any AEs leading to discontinuation | | | | **Range: 0.00%-1.90%** | |
|  | Kuo 2022 | 0 | 37 | 0.00% | 52 weeks |
|  | Yan 2023 | 1 | 74 | 1.35% | 14 weeks |
|  | Lu 2022 | 1 | 65 | 1.54% | 14 weeks |
|  | Chen 2023 | 4 | 211 | 1.90% | 72 weeks |
| Specific reasons for discontinuation | | | | | |
| Enlarged pulmonary nodule | | | | **Range: 1.60%** | |
|  | Huang 2023 | 1 | 64 | 1.60% | 52 weeks |
| Recurrence of symptoms of intestinal aeromonas infection | | | | **Range: 1.23%** | |
|  | Tang 2023 | 1 | 81 | 1.23% | 52 weeks |
| **TRAE** | | | | **Range: 13.90%** | |
|  | Qiao 2023 | 5 | 36 | 13.90% | 30 weeks |
| **Mild TRAE** | | | | **Range: 16.22%** | |
|  | Kuo 2022 | 6 | 37 | 16.22% | 52 weeks |
| **STRAE** | | | | **Range: 0.00%** | |
|  | Kuo 2022 | 0 | 37 | 0.00% | 52 weeks |
| **Serious infection** | | | | | |
| Serious infection | | | | **Range: 0.00%** | |
|  | Tian 2022 | 0 | 24 | 0.00% | 14 weeks |
| Listeria monocytogenes sepsis | | | | **Range: 2.70%** | |
|  | Kuo 2022 | 1 | 37 | 2.70% | 52 weeks |
| **CDI** | | | | **Range: 12.35%** | |
|  | Tang 2023 | 10 | 81 | 12.35% | 52 weeks |
| **Opportunistic infection** | | | | | |
| Tuberculosis | | | | **Range: 0.00%** | |
|  | Huang 2023 | 0 | 64 | 0.00% | Huang 2023 |
|  | Kuo 2022 | 0 | 37 | 0.00% | Kuo 2022 |
|  | Tang 2023 | 0 | 81 | 0.00% | Tang 2023 |
|  | Tian 2022 | 0 | 24 | 0.00% | Tian 2022 |
| Hepatitis B virus infection | | | | **Range: 0.00%** | |
|  | Huang 2023 | 0 | 64 | 0.00% | Huang 2023 |
|  | Kuo 2022 | 0 | 37 | 0.00% | Kuo 2022 |
|  | Tang 2023 | 0 | 81 | 0.00% | Tang 2023 |
| Aeromonas intestinalis infection | | | | **Range: 1.23%** | |
|  | Tang 2023 | 1 | 81 | 1.23% | 52 weeks |
| Cytomegalovirus infection | | | | **Range: 0.00%3.33%** | |
|  | Sun 2023 | 1 | 30 | 3.33% | 14 weeks |
|  | Tian 2022 | 0 | 24 | 0.00% | 14 weeks |
| Herpes zoster | | | | **Range: 2.78%** | |
|  | Qiao 2023 | 1 | 36 | 2.78% | 30 weeks |
| **Infusion reaction** | | | | **Range: 0.00%** | |
|  | Huang 2023 | 0 | 64 | 0.00% | 52 weeks |
|  | Sun 2023 | 0 | 30 | 0.00% | 14 weeks |
|  | Tang 2023 | 0 | 81 | 0.00% | 52 weeks |
|  | Tian 2022 | 0 | 24 | 0.00% | 14 weeks |
| *AE, adverse event; CDI, clostridium difficile infection; SAE, serious adverse events; STRAE, serious treatment-related adverse events; TRAE, treatment-related adverse events* | | | | | |

**Forest plots for efficacy in Asian patients with Crohn’s Disease and Ulcerative Colitis during induction phase**

Fig. S 1 Clinical response rate during induction phase for Asian patients with Crohn’s Disease

Fig. S 2 Clinical response rate during induction phase for Asian patients with Ulcerative Colitis

Fig. S 3 Corticosteroid-free clinical remission rate during induction phase for Asian patients with Ulcerative Colitis

Fig. S 4 Endoscopic remission rate during induction phase for Asian patients with Crohn’s Disease

Fig. S 5 Endoscopic response rate during induction phase for Asian patients with Crohn’s Disease

Fig. S 6 Endoscopic remission rate during induction phase for Asian patients with Ulcerative Colitis

Fig. S 7 Endoscopic response rate during induction phase for Asian patients with Ulcerative Colitis

**Forest plots for efficacy in Asian patients with Crohn’s Disease and Ulcerative Colitis in medium term**

Fig. S 8 Clinical response rate in medium term for Asian patients with Crohn’s Disease

Fig. S 9 Clinical response rate in the medium term for Asian patients with Ulcerative Colitis

Fig. S 10 Corticosteroid-free clinical remission rate in medium term for Asian patients with Crohn’s Disease

Fig. S 11 Endoscopic remission rate in medium term for Asian patients with Crohn’s Disease

Fig. S 12 Endoscopic remission rate in the medium term for Asian patients with Ulcerative Colitis

Fig. S 13 Endoscopic response rate in the medium term for Asian patients with Ulcerative Colitis

**Forest plots for efficacy in Asian patients with Crohn’s Disease and Ulcerative Colitis in long term**

Fig. S 14 Clinical response rate in long term for Asian patients with Crohn’s Disease

Fig. S 15 Clinical response rate in the long term for Asian patients with Ulcerative Colitis

Fig. S 16 Corticosteroid-free clinical remission rate in long term for Asian patients with Crohn’s Disease

Fig. S 17 Corticosteroid-free clinical remission rate in the long term for Asian patients with Ulcerative Colitis

Fig. S 18 Endoscopic remission rate in long term for Asian patients with Crohn’s Disease

Fig. S 19 Endoscopic remission rate in the long term for Asian patients with Ulcerative Colitis
